# Quantitative Analysis of the Spatial Organization of Synaptic Inputs on the Postsynaptic Dendrite

**DOI:** 10.3389/fncir.2018.00039

**Published:** 2018-05-23

**Authors:** Volker Scheuss

**Affiliations:** Department Synapses - Circuits - Plasticity, Max Planck Institute of Neurobiology, Martinsried Germany

**Keywords:** synaptic input, dendritic integration, dendrite, spatial organization, synapse cluster, quantitative analysis

## Abstract

The spatial organization of synaptic inputs on the dendritic tree of cortical neurons is considered to play an important role in the dendritic integration of synaptic activity. Active electrical properties of dendrites and mechanisms of dendritic integration have been studied for a long time. New technological developments are now enabling the characterization of the spatial organization of synaptic inputs on dendrites. However, quantitative methods for the analysis of such data are lacking. In order to place cluster parameters into the framework of dendritic integration and synaptic summation, these parameters need to be assessed rigorously in a quantitative manner. Here I present an approach for the analysis of synaptic input clusters on the dendritic tree that is based on combinatorial analysis of the likelihoods to observe specific input arrangements. This approach is superior to the commonly applied analysis of nearest neighbor distances between synaptic inputs comparing their distribution to simulations with random reshuffling or bootstrapping. First, the new approach yields exact likelihood values rather than approximate numbers obtained from simulations. Second and more importantly, the new approach identifies individual clusters and thereby allows to quantify and characterize individual cluster properties.

## Introduction

Together with the specific connectivity of neurons within a neural circuit and the dynamic properties of their synapses, dendritic computations are considered to play an important role in information processing. Furthermore, active dendritic properties have been suggested to increase the memory storage capacity of neural circuits by structural plasticity (Poirazi and Mel, 2001).

Dendritic integration determines the arithmetic of synaptic summation that translates spatio-temporal patterns of synaptic input into the spiking output of neurons. Dendritic integration and the underlying mechanisms have been characterized using for example direct stimulation of postsynaptic receptors at defined sets of synapses with 2-photon glutamate uncaging in form of systematically varied spatiotemporal patterns of stimulation (e.g., Losonczy and Magee, 2006; Branco and Hausser, 2011). Moreover, it has been shown, that neural computations depend on active dendritic properties *in vivo* (Lavzin et al., 2012; Smith et al., 2013). However, little is known about the spatial organization of synaptic inputs on the dendritic tree of cortical neurons although it is considered to play a central role in dendritic integration. Thus, to understand how the rules of dendritic integration as studied without any knowledge about the origin or type of the stimulated synapses (e.g., Poirazi and Mel, 2001; Losonczy and Magee, 2006; Branco and Hausser, 2011) translate into neural processing, requires to know how specific types of connections are arranged and combined on the postsynaptic dendritic tree. Only recently data on the spatial arrangement of synapses on the dendrites of hippocampal or cortical pyramidal cells and other neurons became available: Functionally defined inputs, i.e., spontaneously active or activated by sensory stimulation, have been identified and mapped using calcium imaging (e.g., Chen et al., 2011; Kleindienst et al., 2011; Takahashi et al., 2012). Genetically and anatomically defined inputs have been mapped using GFP reconstitution across synaptic partners (GRASP, Druckmann et al., 2014), array tomography (Rah et al., 2013) or standard light and electron microscopy (McBride et al., 2008; da Costa and Martin, 2011). More recently, we and others used optogenetics and calcium imaging to identify and map functional synapses arising from a genetically and anatomically defined input (Little and Carter, 2012; Macaskill et al., 2012; Gökçe et al., 2016). Furthermore, calcium imaging *in vivo* has been used to identify cohorts of dendritic spines responsive to specific sensory stimuli (Jia et al., 2010; Chen et al., 2011; Varga et al., 2011; Wilson et al., 2016; Iacaruso et al., 2017; Scholl et al., 2017). Apart from dendritic integration, synapse clusters are also implicated in plasticity underlying learning and memory formation (e.g., Govindarajan et al., 2006; DeBello, 2008; Makino and Malinow, 2011; Fu et al., 2012).

While powerful methods have been developed for neural circuit analysis at the level of cell-to-cell connectivity (e.g., Bassett and Sporns, 2017; Schroter et al., 2017), methods for the systematic analysis of the spatial patterns of the synaptic organization on dendrites are still in their infancy. This is despite the importance of such methods for putting the spatial rules of synaptic organization into the context of the rules for dendritic integration (e.g., Losonczy and Magee, 2006; Branco and Hausser, 2011) and for testing predictions on synapse clustering during plasticity and memory formation from theoretical work (e.g., Kastellakis et al., 2016). To this end, it is required to go beyond the classical analysis of pairwise nearest neighbor distances (e.g., McBride et al., 2008; da Costa and Martin, 2011; Rah et al., 2013; Druckmann et al., 2014) by identifying and characterizing individual clusters.

Here I present an analytical approach for identifying and characterizing synapse clusters. The approach uses a combinatorial analysis of all theoretically possible synapse arrangements for classifying recorded arrangements to be clusters based on their likelihood to occur. This has the advantage over the commonly applied pairwise analysis of nearest neighbors with reshuffling and bootstrapping that it yields exact likelihood values and allows to identify and subsequently characterize individual clusters containing multiple synapses and not just pairs.

## Methods

The coefficient of variation (CV) for the likelihood estimates obtained by random reshuffling (**Figure 4B**) was determined using binomial statistics without the need for performing simulations. If a particular pattern occurs with the probability *p* then it is expected to occur within *n* runs of random reshuffling *n*·*p* times and the estimated likelihood is on average *L* = *n*·*p/n*. The variability in the outcome from run to run is described by the variance of the number of times the pattern is observed in n runs of reshuffling:

(M1) Var(n)=n·p·(1−p)

And thus the *CV*_*L*_ of the estimated likelihood from *n* runs of reshuffling is

(M2) $$CV_{L}=\frac{\sqrt{Var(n)}}{n\cdot p}=\sqrt{\frac{1-p}{n\cdot p}}$$

The computation time required for the combinatorial approach and to run simulations with reshuffling was tested with MATLAB code (see Supplementary Material; MATLAB version R2013b; MathWorks, Natick, MA, USA) on a standard desktop computer (dual core processor, 2.13 GHz, 8 GB RAM, Windows 7 64-bit operating system).

## Results

**Figure 1:**
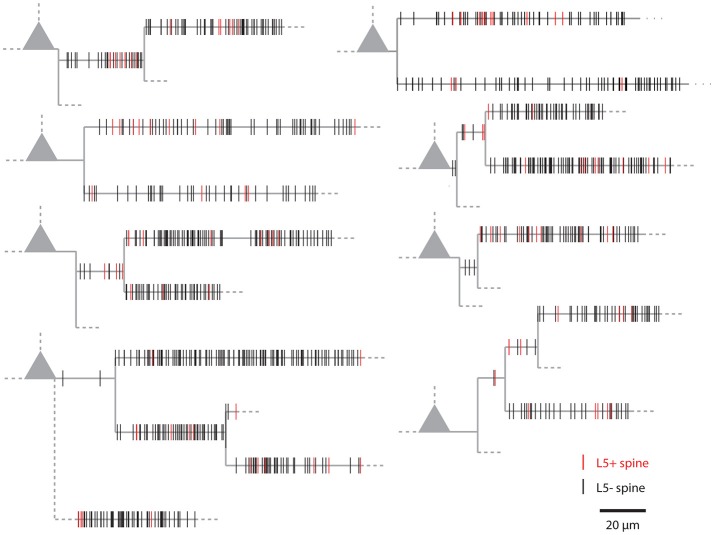
Examples of the spatial organization of synaptic inputs on dendrograms. Example dendrograms of layer 5 pyramidal neurons in mouse primary visual cortex, where spines receiving synapses originating from neighboring layer 5 pyramidal cells have been mapped (red; data from Gökçe et al., 2016).

**Figure 2:**
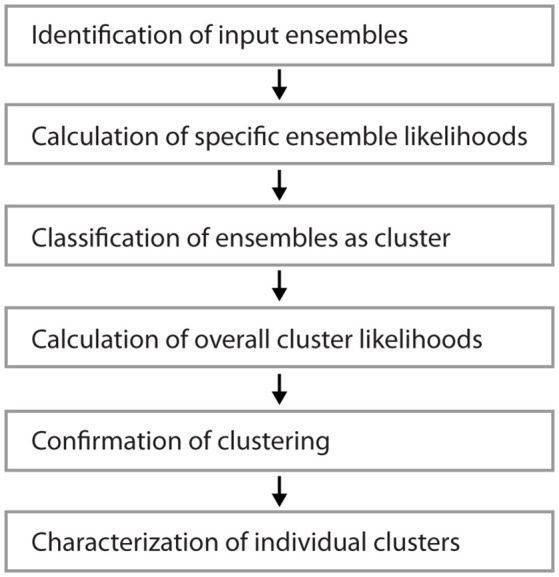
Workflow for the analysis of input clusters. The analysis proceeds in 6 steps that are outlined in the text. An example of its application and its results for one segment and input cluster is given in **Figure 8**.

**Figure 3:**
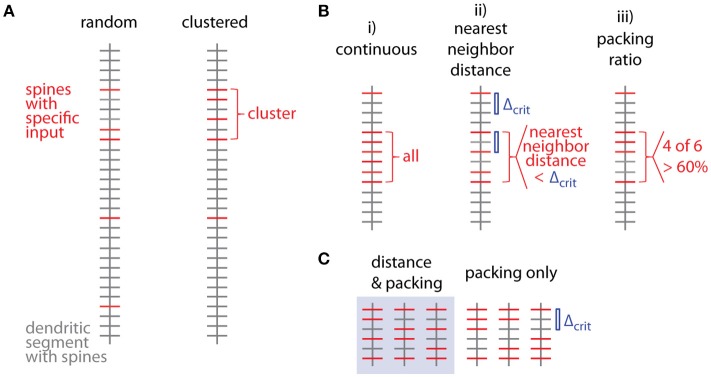
Spatial organization of synaptic inputs on a dendritic segment and cluster criteria. **(A)** Examples of random distributed and clustered spatial organization of synapses of a specific type of input (red) on a dendritic segment. **(B)** Spatial criteria to define ensembles of synaptic inputs. **(C)** Comparison of *nearest neighbor distance* criterion and *packing ratio* criterion. All possible ways of distributing two additional inputs among four spines in between the two specific inputs delimiting the given input ensemble. While the packing ratio (4 out of 6) is the same for all cases, the *nearest neighbor distance* criterion Δ_crit_ (blue bar) is only fulfilled for the 3 cases in the gray box on the left.

Figure 1 shows examples of dendrograms of layer 5 pyramidal cells where the spines targeted by inputs originating from neighboring layer 5 pyramidal cells have been mapped (red spines; Gökçe et al., 2016). Here, a quantitative approach is described for analyzing the spatial organization of inputs on the dendrite. Particular focus is put on testing for the presence of a clustered distribution and identifying individual input clusters. **Figure 3A** shows schematically examples for a random distributed and a clustered spatial organization. The non-random clustered arrangement is defined by specific characteristics, which is a concentration or accumulation of inputs within a certain stretch of dendrite. Nevertheless, even in the case of a random distribution patterns can arise by chance, which resemble non-random patterns. Thus to decide whether or not the spatial organization is random or non-random one needs to compare the probability of observation of the patterns found in the data vs. the likelihood of occurrence of those patterns in case of a random distribution. The cluster detection and analysis proceeds in 6 steps (see workflow in Figure 2 and example in **Figure 8**):

1. Identification of input ensembles based on the ensemble criterion
2. Calculation of the *specific ensemble likelihood* for the particular ensembles within the dendritic segments or branched trees
3. Classification of ensembles as cluster according to ensemble likelihood criterion
4. Calculation of the *overall cluster likelihood* for any type of cluster to occur in the dendritic segments or branched trees
5. Comparison of the observed and expected numbers of dendritic segments or branched trees with an input cluster
6. Analysis of parameters and characteristics of individual clusters

These steps will be discussed in the following. For clarity the mathematical concepts will be developed first for what I call the *order based case*, where synapses/inputs are simply counted along the dendrite. In other words, this is the special case of uniform nearest neighbor distance where there is no need for explicitly taking this distance into account. The transformation to the *distance based case*, where the real non-uniform distances between synapses/inputs are taken into account, will be introduced later. The derivation assumes that all synapse locations, i.e., spine positions, are defined and the assignment of input categories to the given synapses is known. In real data this is not necessarily the case, first because synapse location and input identities are often mapped only for part of the dendritic tree and second, because a fraction of spines/synapses might remain undetected in imaging systems and therefore unaccounted for. If only part of a dendritic tree can be mapped in one experiment, the best approach is to roughly map the global input distribution and then selecting for detailed mapping those areas with significant input density or those of particular interest. Subsequently, separate experiments are performed on sets of cells for each area to be mapped. In order to be able to consider branched trees up to the whole dendritic tree of a neuron, the original approach restricted to individual segments as presented in short form in Gökçe et al. (2016) is extended below to branched trees. The fraction of undetected synapses and inputs due to technical reasons should be low. It is reasonable to assume that the distribution of missed synapses/inputs is uniform along the dendrite and therefore does not significantly affect the results.

### Step 1: identification of input ensembles based on the ensemble criterion

Several criteria can be used to define ensembles of inputs (Figure 3B):

- A “continuous” sequence of directly neighboring inputs, which are all of the same type.
- An ensemble with distance from any input to its nearest neighbor of the same type ≤ threshold (e.g., 10 μm, Takahashi et al., 2012).
- An ensemble with packing ratio (ratio of number of inputs of one type over the total number of inputs) ≥ threshold (e.g., 60%).

Criterion (i) is more a theoretical definition with little practical/biological relevance (but see Fu et al., 2012 for the case of clustered spine formation). Criterion (ii) is weaker than criterion (i) by allowing “gaps” between inputs specified by an upper limit on the nearest neighbor distance. Biologically this distance can be justified by the length constant for the considered interaction between neighboring inputs such as electrical interactions during dendritic integration (Losonczy and Magee, 2006; Branco and Hausser, 2011) or biochemical interactions during plasticity (Harvey and Svoboda, 2007). This is a criterion which has been applied in conventional cluster analysis based on nearest neighbor distances (e.g., Takahashi et al., 2012). Criterion (iii) is again a weaker version of criterion (ii): Their relationship is obtained by considering, that inputs spaced at the maximum distance satisfying the distance criterion Δ_*crit*_ (criterion ii) would satisfy also the lowest accepted packing ratio PR_crit_ (criterion iii). In this case *M* = *m*·Δ_*crit*_ + 1, where *M* is the total number and *m* the number of spines with the input of interest in the ensemble (see Figure 3C; compare also **Figure 5D** for analogous relationship between *M* and *n*). With the packing ratio defined as ratio of the number of inputs m over all *M* spines in an ensemble, this yields:

(1) $$PR_{crit}=\frac{m}{M}=\frac{m}{m\cdot \Delta_{crit}+1}\approx \frac{1}{\Delta_{crit}}\text{ for large }m$$

Thus the packing ratio threshold and nearest neighbor distance criterion are inversely related. However, it has to be noted that packing ratio and nearest neighbor distance criterion are not completely equivalent. For the packing ratio there is no requirement on the internal arrangement of inputs while the nearest neighbor distance criterion places an upper bound on the gaps within an ensemble (Figure 3C). Here and in the following steps, criterion (ii) is used to define input ensembles.

### Step 2: calculation of the *specific ensemble likelihood* on individual segments

**Figure 4:**
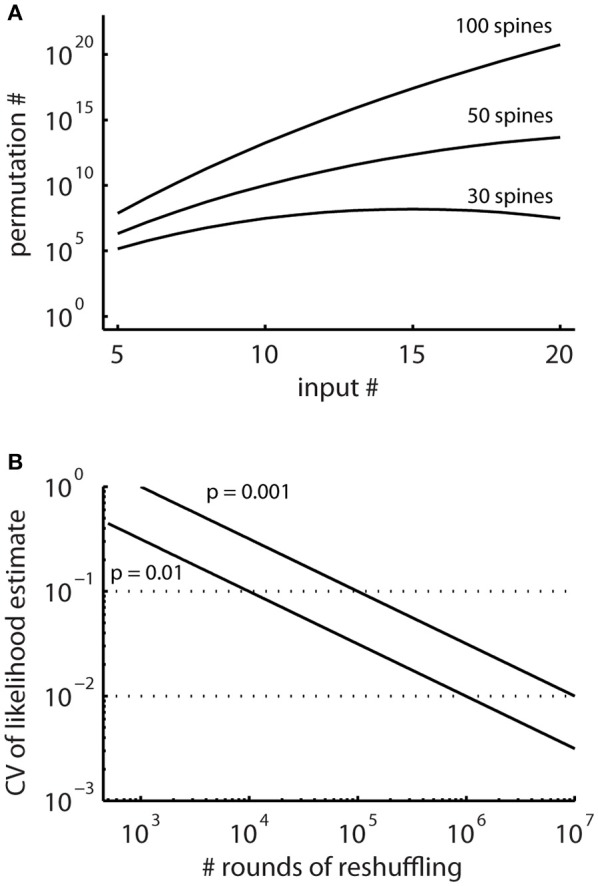
The total numbers of all possible spatial arrangements of synaptic inputs are large. **(A)** Numbers of possible permutations, when assigning increasing number of synaptic inputs to 30, 50, and 100 spines calculated with Equation (2). **(B)** Coefficient of variation (CV, Equation M2) of probability estimates obtained by simulations where surrogate distributions are generated with random reshuffling. This describes the expected error in the estimated likelihoods relative to the number of simulation runs.

In principle, running the cluster detection on the surrogate data generated by random reshuffling provides an estimate of the probability of occurrence of clusters (e.g., Takahashi et al., 2012; Yadav et al., 2012; McBride and DeBello, 2015). However, the number of possible arrangements can be very large (Figure 4A) and requires large numbers of reshuffling rounds. The advantage of the combinatorial approach over estimating likelihoods using random reshuffling is that it provides exact values for the small likelihoods involved, for which reliable estimates would require large numbers of rounds of reshuffling.

The total number of patterns of assigning *n* responsive spines to *N* total spines is

(2) $$\left(\begin{array}{c} N\\ n \end{array}\right)$$

In particular, for patterns with low likelihood, which are the patterns of interest, the number of reshuffling rounds required for determining their likelihood with sufficient accuracy are very large in reshuffling simulations, where the inputs are randomly assigned to the present synapses (Figure 4B). Therefore, I derive together with the concept for characterizing ensembles as clusters an analytical solution for determining ensembles likelihoods based on combinatorial analysis.

**Figure 5:**
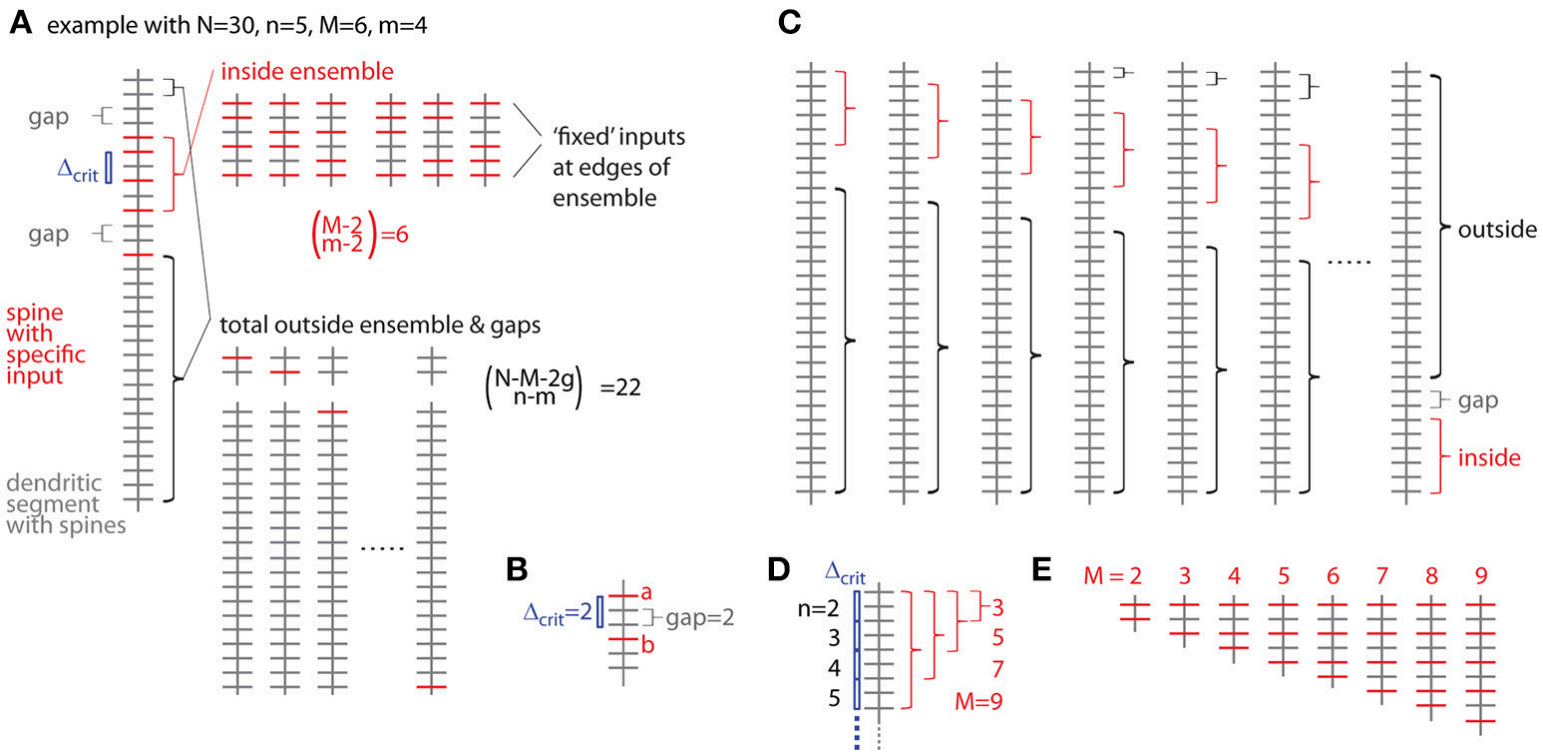
Combinatorial analysis of input ensemble likelihood. **(A)** Left, one example of a dendritic segment with *N* = 30 spines. *n* = 5 spines receive input from a specific presynaptic population, *m* = 4 of which are part of an input ensemble of size *M* = 6. The ensemble is flanked by gaps of *g* = 2 spines, which do not receive the specific input. Top right, all possible arrangements of *m* = 4 inputs in an ensemble of *M* = 6 spines. Bottom right, all possible arrangements of the remaining *n-m* = 1 inputs over the spines outside ensemble and leading as well as trailing edge. **(B)** Relationship between nearest neighbor distance criterion and gap parameter Δ_crit_ = gap, exemplified for Δ_crit_ = gap = 2: The distance between spine a and b is just above the nearest neighbor distance criterion of Δ_crit_ = 2 and the gap between them in terms of spines is gap = 2. **(C)** All possibilities to place the ensemble of size *M* = 6 into the dendritic segment with *N* = 30. At positions close to the beginning and end of the segment, the leading or trailing gap, respectively, are not fully realized. In the extreme cases, when the ensemble is positioned at the beginning or the end of the segment, no leading or trailing edge, respectively, is necessary to delineate the ensemble. In these cases with reduced gap sizes, the number of spines corresponding to the difference between regular and reduced gap size add to the number of spines outside ensemble and gaps over which the inputs are distributed that are not contained in the ensemble. **(D)** Example of the dependence of the maximal cluster size M on the total number of inputs n for a gap/nearest neighbor distance criterion of 2. **(E)** Examples of all ensemble types possible with a total of *n* = 5 inputs and a gap/nearest neighbor distance criterion of 2.

The probability of occurrence can be calculated by determining all possibilities of occurrences of patterns/clusters with a similar metric and dividing their number by the total number of all possibilities to assign the given number of inputs *n* to the total number of spines/synapses *N*. The most intuitive metric is the total number of spines/synapses in the ensemble *M*, i.e., the cluster size, and the number of inputs in the ensemble *m*. Furthermore a gap *g* has to be specified, which describes to what extend the ensemble should be separated from any other input outside the ensemble (Figure 5A). The gap parameter *g* corresponds to the nearest neighbor distance criterion (see above and Figure 5B). The number of ways to place *m* responsive spines into an ensemble of size *M* is (Figure 5A):

(3a) $$\left(\begin{array}{c} M\\ m \end{array}\right)$$

But we need to exclude empty edges, i.e., the first and last spine in the ensemble must receive the specific input. This is like distributing *m–*2 over *M–*2 slots (Figure 5A), thus

(3b) $$\left(\begin{array}{c} M-2\\ m-2 \end{array}\right)$$

The number of ways for assigning the remaining *n-m* inputs to the remaining *N–M* − *2 g* spines outside the sensemble enforcing a leading and trailing gap (Figure 5A) is:

(4) $$\left(\begin{array}{c} N-M-2g\\ n-m \end{array}\right)$$

The product of Equations (3b) and (4) provides the number of ways for assigning inputs to spines with the ensemble being located in one particular position along the dendrite. However, at least with respect to combinatorial statistics I assume that all possible positions are equivalent. The obvious solution would then be to multiply Equations (3) and (4) with the number of locations a patch of *M* synapses can be placed onto a dendritic segment with *N* total synapses (Figure 5C)

(5) N−M+1

However, this is not completely correct, because at patch locations close to the ends of the dendritic segment the numbers of synapses to remain unoccupied are smaller than the required gap size and this difference has to be taken into account with respect to the number of synapses, which can be assigned with inputs outside of the ensemble (Figure 5C):

(6) $$2\underset{i\;=\;0}{\sum^{g\;-\;1}}\left(\begin{array}{c} N-M-g-i\\ n-m \end{array}\right)+\left(N-M-2g+1\right)\left(\begin{array}{c} N-M-2g\\ n-m \end{array}\right)$$

Here the first term sums those cases one by one, where leading or trailing gap consist of fewer synapses than the gap size, in other words, where none of the *n-m* inputs can be placed at the beginning of the dendritic segment in front of or at the end behind the ensemble patch, respectively. The second term describes the remaining *N – M* - 2*g* + 1 cases where full leading and trailing gaps have to be considered (see Figure 5C). Thus the likelihood to find an ensemble with *M* and *m* is the product of Equations (3b) and (6) divided by Equation (2):

(7) $$\begin{array}{l} p_{N,n}\left(M,m,g\right)=(2\underset{i\;=\;0}{\sum^{g\;-\;1}}\left(\begin{array}{c} N-M-g-i\\ n-m \end{array}\right)\\ \;+\;(N-M-2g+1)\left(\begin{array}{c} N-M-2g\\ n-m \end{array}\right))\frac{\left(\begin{array}{c} M-2\\ m-2 \end{array}\right)}{\left(\begin{array}{c} N\\ n \end{array}\right)} \end{array}$$

I now define an ensemble type as the subset of ensembles having M synapses, of which m or more receive input from the presynaptic population of interest. The rationale behind this is that any specific biological mechanisms that depends on the number of clustered inputs can be assumed non-operational with fewer but operational with more inputs than the particular number. The likelihood to observe a particular ensemble type, which I refer to as *specific ensemble likelihood* (*SEL*), is then

(8) $$SEL_{N,n}\left(M,m,g\right)=\underset{i\;=\;m}{\sum^{\text{min}\left(n,M\right)}}p_{N,n}\left(M,i,g\right)$$

### Alternative step 2: calculation of the *Specific Ensemble Likelihood* for branched trees

This section concerns the extension of the approach from analyzing individual dendritic segments to analyzing a branched tree of segments up to the whole dendritic tree of a neuron. This extension comprises a modification of Equation (7) for the likelihood for finding an ensemble within an individual segment of the tree and in addition calculating the likelihood for finding ensembles located on the individual branching points between the segments.

**Figure 6:**
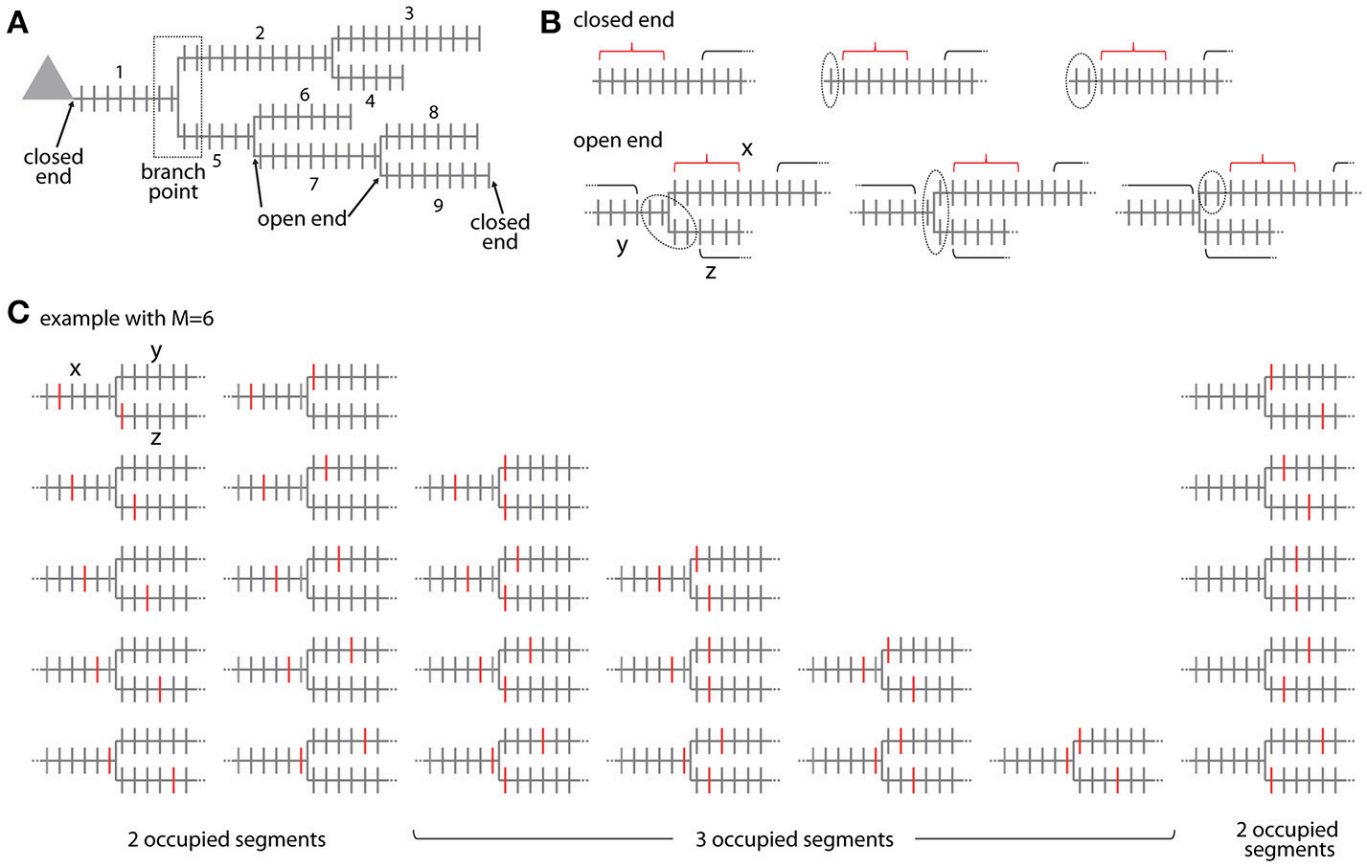
Combinatorial analysis of input ensemble likelihood in branched trees. **(A)** Example of a branched tree of segments. Segment number 1 has a *closed end* at the soma and segments number 3, 4, 6, 8, and 9 at their terminal ends. All other segments have *open ends* on both sides. The branch point between segment numbers 1, 2, and 5 is indicated by a dotted square. **(B)** Arrangement of the gaps delimiting an ensemble in the *closed end* (top) and *open end* condition (bottom). Example with ensemble size *M* = 6 and gap size *g* = 2. Red bracket, ensemble; black bracket, spines outside ensemble and gaps; dotted line, gap. In the *open end* condition the gap extends beyond segment x to the adjacent segments y and z. **(C)** Example of all possible placements of an ensemble of size *M* = 6 at a branch point. Ensembles are marked here by the spines that receive the specific input at the edges of the ensemble. Note the cases where the ensembles cover only two or all three segments.

The total number *N*_*tot*_ of spines across all segments in the tree is

(9) $$N_{tot}=\sum_{x}N_{x}$$

where the index *x* denotes the branch indices as defined in Figure 6A. Likewise the total number *n*_*tot*_ of inputs on the tree is

(10) $$n_{tot}=\sum_{x}n_{x}$$

For calculating the likelihood to find an ensemble in an individual segment of the tree in analogy to Equation (7) requires distinguishing two cases: The segment arising at the soma and the terminal segments have at one end a branch point and at the other end terminate without any further synapses existing beyond that point (e.g., segments *x* = 1, 3, 4, 6, 8, 9 in Figure 6A). All other segments lie between two branch points such that further synapses exist beyond their endpoints (e.g., segments *x* = 2, 5, 7 in Figure 6A). These synapses have to be taken into account when determining the leading or trailing gap of ensembles located close to the segment ends. I term the end of a segment, where no further synapses existing beyond that point *closed end*. Examples are shown in Figure 6B top row. The *closed end* condition corresponds to the leading and trailing gap conditions in the treatment of individual segments (section Step 2: Calculation of the *Specific Ensemble Likelihood* on Individual Segments) and is contained in the sum in the first term of Equation (7) that describes the number of ways inputs can be arranged outside ensemble and gaps. In the other case, I term the end of a segment *open end*, where further synapses exist beyond that point on the adjacent segments at the given branch point. Examples are shown in Figure 6B bottom row. In the *open end* condition the gap has to extend into both adjacent segments to a degree that depends on how close the ensemble is located to the end point of the segment. If *i* spines of the gap of size *g* are located on the segment itself then gaps with *g-i* spines have to be located on each of the adjacent segments. The number of spines that are part of the gap is therefore:

2(g−i)+i=2g−i

To arrive at the number of ways inputs can be arranged outside ensemble and gaps in the *open end* case, this equation replaces the stepwise reduced number *g-i* of gap spines in the sum in the first term of Equation (7). Thus the likelihood $p_{x}^{s}$ to find an ensemble with *M* and *m* on segment *x* is in analogy to Equation (7) with all inputs not contained in the ensemble being distributed over the whole tree outside ensemble and gaps:

(11) $$\begin{array}{l} p_{x}^{s}\left(M,m,g\right)\\ \;=\;(\delta_{x}^{closed\;end}\;\underset{i=0}{\sum^{g-1}}\left(\begin{array}{c} N_{tot}-M-g-i\\ n_{tot}-m \end{array}\right)\\ \;+\;\delta_{x}^{open\;end}\;\underset{i=0}{\sum^{g-1}}\left(\begin{array}{c} N_{tot}-M-2g+i\\ n_{tot}-m \end{array}\right)\\ \;+\;(N_{x}-M-2+1)\left(\begin{array}{c} N_{tot}-M-2g\\ n_{tot}-m \end{array}\right))\frac{\left(\begin{array}{c} M-2\\ m-2 \end{array}\right)}{\left(\begin{array}{c} N_{tot}\\ n_{tot} \end{array}\right)} \end{array}$$

with

$$\delta_{x}^{closed\;end}=\{\begin{array}{cc} 0 & \text{if dendritic segment is not an initial/terminal segment}\\ 1 & \text{if dendritic segment is an initial/terminal segment} \end{array}$$

and

$$\delta_{x}^{open\;end}=\{\begin{array}{cc} 1 & \text{if dendritic segment is an initial/terminal segment}\\ 2 & \text{if dendritic segment is not an initial/terminal segment} \end{array}$$

It has to be noted, that Equation (11) assumes in the *open end* condition that the gap extending onto adjacent segments is completely contained in these segments and does not extend further to the next segments, i.e., for the number of spines *N*_*y*_ and *N*_*z*_ on the adjacent segments holds *N*_*y*_ ≥ *g* and *N*_*z*_ ≥ *g*. However, if gaps would have to extend to further segments Equation (11) would still yield an upper estimate of the likelihood, because the fraction of the gap extending further would cover two segments instead of one such that the number of ways of distributing the synapses outside of the ensemble would be reduced. Treating such special cases explicitly in Equation (11) would be complex and blur the presentation of the principle. Nevertheless, algorithms for calculation the likelihoods from data should account for such cases.

Ensembles located at branch points occupy at least two or all three adjacent segments. The ensemble definition at a branch point is as defined for ensembles along a segment that the ensemble contains *M* spines in total. The cases that the given ensemble covers only two or all three segments have to be distinguished. If the ensemble spans two segments, it has two ends and the first and last spine need to receive the specific input as in Equation (3b). The number of ways an ensemble of size *M* can span two segments at a branch point is given by (see example with *M* = 6 in Figure 6C)

2(M−1)+M−1=3(M−1)

This is derived by considering that there are *M*−*1* ways to place an ensemble of size *M* on a segment such that ≥1 spines are located on one of the adjacent segments and multiplying this by two since there are two adjacent segments (first term on left hand side). In addition there are *M*−*1* ways for placing the ensemble on these two segments alone (second term on left hand side). If the ensemble spans three segments, it has three ends and three spines, one at each end, need to receive the specific input in analogy to Equation (3b). The number of ways an ensemble of size *M* can span three segments at a branch point is given by the binomial coefficient (see example with *M* = 6 in Figure 6C)

$$\left(\begin{array}{c} M\\ 3 \end{array}\right)=\frac{M!}{3!\left(M-3\right)!}=\frac{(M-2)\left(M-1\right)}{2}$$

The likelihood $p_{x,y,z}^{bp}$ to find an ensemble with parameters *M* and *m* at the branch point between segments *x, y, z* is with all inputs not contained in the ensemble and gaps being distributed over the whole tree outside ensemble and gaps:

(12) $$\begin{array}{l} p_{x,y,z}^{bp}\left(M,m,g\right)=(3\left(M-1\right)\left(\begin{array}{c} M-2\\ m-2 \end{array}\right)\\ \;+\;\frac{(M-2)\left(M-1\right)}{2}\left(\begin{array}{c} M-3\\ m-3 \end{array}\right))\frac{\left(\begin{array}{c} N_{tot}-M-3g\\ n_{tot}-m \end{array}\right)}{\left(\begin{array}{c} N_{tot}\\ n_{tot} \end{array}\right)} \end{array}$$

The first term in the sum of Equation (12) covers the case of two occupied segments and the second term the case of three occupied segments. In any case three gaps, one on each of the adjacent segments, are required, such that the inputs not part of the ensemble have to be distributed over all spines outside the ensemble and three gaps (compare Equation 6). Similar to Equation (11) as discussed above, Equation (12) assumes that the ensemble and the adjoining gaps are completely contained in the segments at the branch point and do not extend via neighboring branch points to the next segments. Algorithms for calculating the likelihoods from data should account for such cases.

Summation of Equations (11) and (12) over all segments and branch points yields the likelihood to find an ensemble with *M* and *m* on the tree:

(13) $$p_{N_{tot},n_{tot}}^{tree}\left(M,m,g\right)=\sum_{x}p_{x}^{s}\left(M,m,g\right)+\sum_{xyz}p_{x,y,z}^{bp}\left(M,m,g\right)$$

In analogy to Equation (8) the specific ensemble likelihood within the tree is given by

(14) $$SEL_{N_{tot},n_{tot}}^{tree}\left(M,m,g\right)=\underset{i=m}{\sum^{\text{min}\left(n_{tot},M\right)}}p_{N_{tot},n_{tot}}^{tree}\left(M,i,g\right)$$

### Step 3: classification of ensembles as cluster according to ensemble likelihood criterion

In this step, ensembles are classified as clusters if their *specific ensemble likelihood* is below or equal to an empirical upper likelihood threshold. The rationale is that local aggregations of inputs are considered as clusters only if their probability to occur by chance is low. In Gökce et al., we used an upper likelihood threshold of 1% to classify input ensembles as clusters. In a similar approach a likelihood threshold of 2.5% was proposed (Bendels et al., 2010). The appropriateness of the chosen likelihood threshold can be tested based on the *overall cluster likelihood* calculation (Step 4) and statistically testing the hypotheses of random vs. clustered input distributions (step 5).

Apart from the likelihood threshold, classification as cluster might be restricted by additional criteria. For example, the additional rules can be imposed, e.g., that clusters have to contain at least a certain number of inputs, e.g., 3 inputs, and have at least a certain size. Furthermore, in cases where ensembles encompasses an entire dendritic segment, these can be excluded from classification as cluster. In other words, subsets of ensembles, which would qualify as cluster based on the *specific ensemble likelihood* threshold, but have parameters *M* and *m* with *N* ≥ *M* ≥ *N*-2*g* and *m* = *n* would be excluded.

### Step 4: calculation of the *overall cluster likelihood*

The forth step is concerned with the likelihood to observe any cluster on a given dendritic segment or branched tree, which I refer to as *overall cluster likelihood* (*OCL*). This likelihood is determined by considering all possible ensemble types, calculating their *specific ensemble likelihoods*, and adding up those that are equal or below the *specific ensemble likelihood* of the cluster actually present:

(15) $$\begin{array}{l} OCL_{N,n}\left(M^{*},m^{*},g\right)\\ \;=\;\sum_{M=2}^{\left(n-1\right)\cdot g+1}\;\;\sum_{m=2}^{\text{min}\left(n,M\right)}SEL_{N,n}\left(M,m,g\right)\cdot \delta_{N,n}\left(M,m,g\right)\\ \delta_{N,n}\left(M,m,g\right)\\ \;=\{\begin{array}{cc} 1 & \text{if }SEL_{N,n}\left(M,m,g\right)\leq \;SEL_{N,n}\left(M^{*},m^{*},g\right)\\ 0 & \begin{array}{l} \;<SEL_{N,n}\left(M,m-1,g\right)\\ \text{ otherwise} \end{array} \end{array} \end{array}$$

For calculating *OCL*_*Ntot, ntot*_ for a branched tree *SEL*_*N, n*_ in Equation (15) is replaced by *SEL*_*Ntot, ntot*_ from Equation (14) and *N* and *n* are replaced by the total number of spines *N*_*tot*_ and inputs *n*_*tot*_, respectively. The upper limit in the outer sum is the largest ensemble size *M* that is possible for any given total number of inputs n and gap size g (see Figure 5D). The factor δ_*N, n*_*(M,m,g)* takes care for not counting any arrangement more than once since the *specific ensemble likelihood* of a particular ensemble type defined by *M* and *m* is the sum of the probabilities over all ensembles with *M* spines and *m, m*+1, *m*+2 … ≤ *M* inputs (Equations 8 or 14). Thus from all *specific ensemble likelihoods* below the cluster criterion for any cluster of size *M* only the largest has to be considered, i.e., the one for the smallest number of inputs. This is because for a given cluster size *M* the *specific ensemble likelihood* decreases with increasing number of inputs m. In Figure 5E, all the possible ensemble types for the example with *N* = 30, *n* = 5 and gap/distance criterion of *g* = 2 are shown. The smallest ensemble has *M* = 2 and the largest, that is possible with *n* = 5 and a gap/distance criterion of 2, has *M* = 9. Table 1 lists the *specific ensemble likelihoods* of these ensembles for different numbers of inputs. Ensembles of *M* = 4 spines with *m* = 4 inputs, of *M* = 5 spines with *m* = 4 or 5 inputs, and of *M* = 6 to 9 spines with *m* = 5 inputs each have *specific ensemble likelihoods* below 1% and thus qualify as clusters. For calculating the *overall cluster likelihood* the *specific ensemble likelihoods* added up over all *M*'s are only those below 1% for the smallest m for each *M* (Table 1, those in bold) in order not to account for any cluster arrangement twice (e.g., in the case of *M* = 5 in Table 1). In the example in Figure 5 and Table 1, the *overall cluster likelihood* is 0.0253 (sum of bold numbers in Table 1).

**Table 1:** Specific ensemble likelihoods for the example in Figure 5 as basis for the calculation of the overall cluster likelihood.

**m\M**	**2**	**3**	**4**	**5**	**6**	**7**	**8**	**9**
2	0.37	0.38	–	–	–	–	–	–
3	–	0.046	0.088	0.12	–	–	–	–
4	–	–	**0.0040**	**0.01**	0.021	0.034	–	–
5	–	–	–	1.8e−04	**7.0e**−**04**	**0.0017**	**0.0033**	**0.0056**

*Bold, ensemble likelihood values ≤ 1% that are added over all M to yield the overall cluster likelihood*.

### Step 5: comparison of the observed and expected numbers of dendritic segments with an input cluster

In the final step, the *overall cluster likelihood* to observe any type of cluster on a given dendritic segment or branched tree is used to calculate the probability to find the number c of observed segments/trees containing a cluster in a data set of *S* analyzed segments/trees. Since the *overall cluster likelihood* in different segments/trees with different types of clusters differ, I use the maximum *overall cluster likelihood OCL*_*max*_ in the data set. The probability to observe exactly *c* segments/trees containing clusters is then given by binomial statistics:

(16) p(c)=(Sc)OCLmax(1−OCLmax)S−cc

The probability to observe at least *c* segments/trees containing a cluster in the data set of *S* segments/trees is then

(17) P=∑Sx=cp(x)=∑Sx=c(Sx)OCLmax(1−OCLmax)S−xx

This represents also the *P* value of the binomial test for the null hypothesis that the number of segments/trees containing a cluster arises from a random distribution (Yadav et al., 2012).

It is possible that individual *overall cluster likelihood* values occur in a dataset that are so high that the probability to observe the given number of segments/trees with a cluster lies above the significance threshold (e.g., Gökçe et al., 2016). In such case, the observation of clusters might still be statistically significant. This can be tested in the following way: The *OCL* values are sorted in ascending order. Then the probability to obtain at least c segments/trees containing a cluster with an *OCL* ≤ *OCLmax(c)* in the data set of S segments/trees is calculated by binomial statistics analogous to Equation (16) with *OCLmax(c)* as upper bound on *OCL*:

(18) $$P(c,OCL_{max})=\underset{x=c}{\sum^{S}}\left(\begin{array}{c} S\\ x \end{array}\right)OCL_{max}{\left(c\right)}^{x}{\left(1-OCL_{max}(c)\right)}^{S-x}$$

Plotting *P* against c shows how many segments/trees with a cluster lie below the significance level for supporting the hypothesis of a clustered non-random distribution.

### Step 6: analysis of parameters and characteristics of individual clusters

Once a clustered distribution is confirmed, the quantitative characterization of the individual identified clusters is performed. The two main parameters are the number of inputs contained in the cluster and the length the cluster extends along the dendrite, which are relevant in the context of dendritic integration and the arithmetic rules of synaptic summation. In addition, the number of total spines/synapses in the cluster, i.e., including both, the spines/synapses with identified and unidentified input, is of interest with respect to the potential combination and integration of the specific input with other inputs. Morphological spine/synapse properties, such as spine head size and neck length, as well as spine/synapse density within compared to outside of clusters are parameters related to structural plasticity and synapse strength. Finally, the location and distance of clusters on the dendrite relative to the soma, and whether they are randomly or systematically distributed, plays a role in dendritic integration.

### Transformation to distance based treatment for the case of individual segments

**Figure 7:**
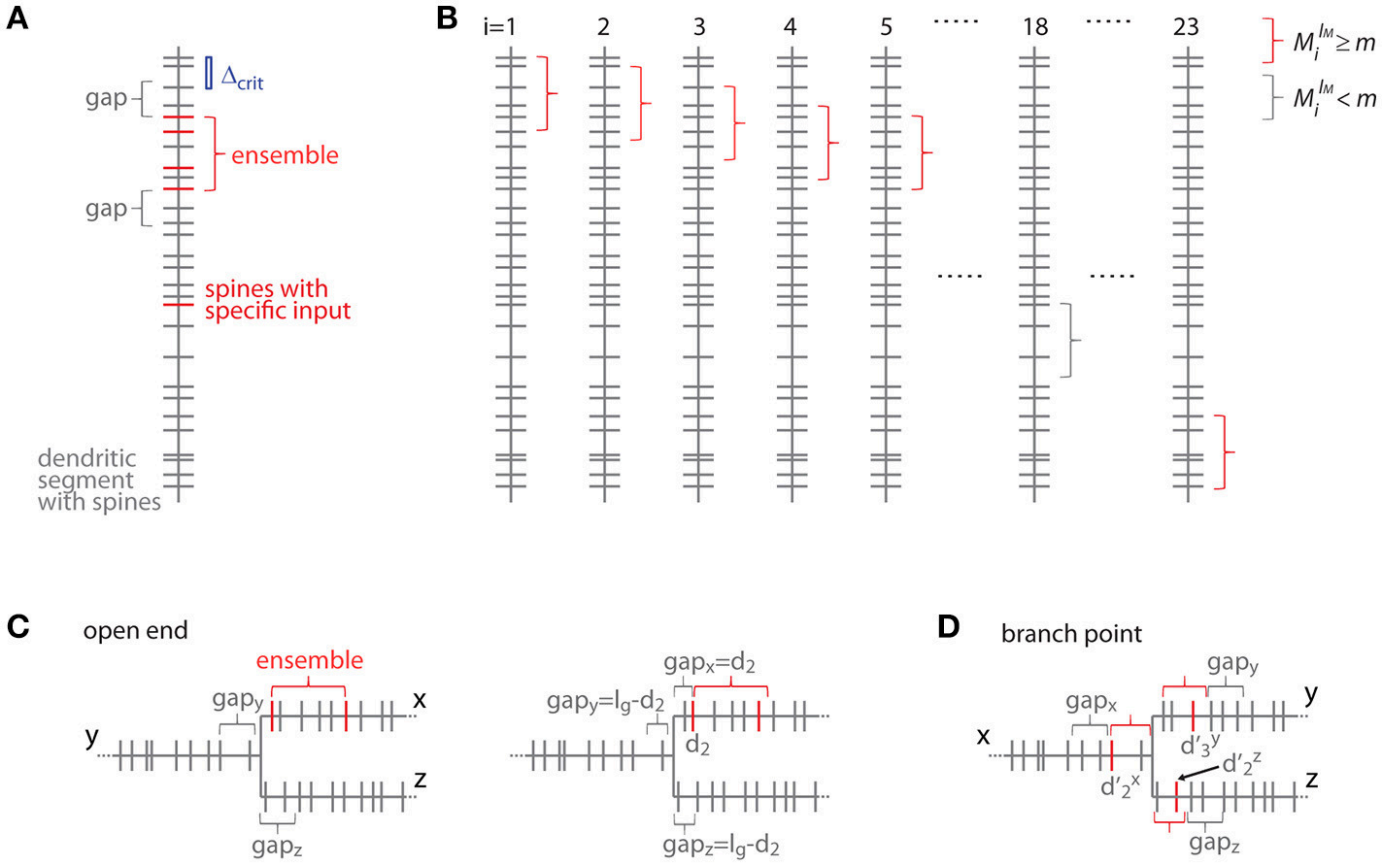
Transformation to the distance based case. **(A)** Example of a dendritic segment with irregularly spaced spines. 5 spines receive the specific input (red), 4 of which are part of an ensemble (red bracket), because they comply with the nearest neighbor distance criterion Δ_crit_ (indicated by the blue bar). The length of the leading and trailing gaps, which flank the ensemble, correspond to the nearest neighbor distance criterion. No spine located within these gaps receives the specific input. **(B)** All possible placements of the given ensemble in **(A)** with length *l*_*M*_ and *m* or more spines onto the dendritic segment. These are obtained by moving the particular ensemble length from spine to spine (i = 1, 2, 3, … 23). Ensembles which fulfill the condition that they contain *m* or more spines are marked with red and others with gray brackets, respectively. **(C)** Illustration of the *open end* condition in the distance based case. Left, situation when the ensemble is located at the beginning of segment x and the gap extends in its full length onto segments y and z. Right, the ensemble on segment x begins with the spine at a distance of d_2_ such that part of the gap is located on segment x and gaps of the remaining length are located on segments y and z. **(D)** Illustration of an ensemble at a branch point in the distance based case. The ensemble occupies all three segments x, y and z. Its total extend, i.e., the summed distances of the limiting spines relative to the branch point d'2x + d'3y + d'2z, has to fulfill the condition of being smaller or equal to the ensemble length l_M_. The ensemble is delimited on each segment by a gap.

For clarity of the mathematical concepts the equations above were developed for what I call the *order based case*, where synapses/inputs are simply counted along the dendrite. In reality, synapses and spines are not uniformly placed along the dendrite, but rather with variable nearest neighbor distances and thus varying density. To take into account the actual distances between synapses, I introduce here the transformation of the above equations to the *distance based case*. The definition of an ensemble type in the *order based case* above was that of a subset of ensembles having *M* synapses, of which m or more receive input from the presynaptic population of interest. Now in the distance based case this definition of ensemble type transfers to the subset of ensembles of length ≤ *l*_*M*_, which contain ≥ *M* spines, of which ≥ *m* receive the specific input. With this definition an equation analogous to Equation (7) can be derived. This is done by determining all positions along the dendritic segment, where the conditions of the ensemble definition are satisfied. This depends on the local spine densities and provides numbers of spines located in the ensemble and in the gaps. Based on these numbers the number of possible arrangements can be calculated. In principle it is like moving along the dendritic segment spine by spine like for Equation (7) but now checking also whether the spatial “constraints” are obeyed, i.e., no responsive or no spines at all in the gaps of certain length before and after an ensemble of certain length with m or more responsive spines (Figure 7):

(19) $$\begin{array}{l} p_{N,n}\left(l_{M},m,l_{g}\right)\\ \;=\;\left(\underset{i=1}{\sum^{N}}\left(\begin{array}{c} M_{i}^{l_{M}}\left(m\right)-2\\ m-2 \end{array}\right)\left(\begin{array}{c} N-M_{i}^{l_{M}}\left(m\right)-g_{i}^{l_{g}}-h_{i}^{l_{g}}\\ n-m \end{array}\right)\right)\frac{1}{\left(\begin{array}{c} N\\ n \end{array}\right)} \end{array}$$

Where the number of spines in an ensemble of length *l*_*M*_ starting with the spine at position *d*_*i*_ is

$$\begin{array}{l} M_{i}^{l_{M}}\left(m\right)=\\ \{\begin{array}{cc} \text{number of spines in}\left[d_{i},d_{i}+l_{M}\right] & \text{if number of spines in }\left[d_{i},d_{i}+l_{M}\right]\geq m\\ 0 & \text{otherwise} \end{array} \end{array}$$

and the number of spines *g* and *h* in the trailing and leading gaps of length *l*_*g*_, respectively, are

$$\begin{array}{l} g_{i}^{l_{g}}=\{\begin{array}{cc} \text{number of spines in}\left[0,d_{i}\right] & \text{if }d_{i}\leq l_{g}\\ \text{number of spines in}\left[d_{i}-l_{g},d_{i}\right] & \text{if }d_{i}>l_{g} \end{array}\\ h_{i}^{l_{g}}=\{\begin{array}{cc} \text{number of spines in}\left[d_{i}+l_{M},d_{i}+l_{M}+l_{g}\right] & \text{if }d_{i}+l_{M}+l_{g}<d_{N}\\ \text{number of spines in}\left[d_{i}+l_{M},d_{N}\right] & \text{if }d_{i}+l_{M}+l_{g}\geq d_{N} \end{array} \end{array}$$

The gap length corresponds to the nearest neighbor distance criterion *l*_*g*_ = Δ_*crit*_ since an ensemble or cluster is delimited by those inputs, of which the prior or following input is further away than the nearest neighbor distance criterion.

With Equation (19) the *specific ensemble likelihood* for the *distance based case* becomes

(20) $$SEL_{N,n}\left(l_{M},m,l_{g}\right)=\underset{i=m}{\sum^{\text{min}\left(n,M\right)}}p_{N,n}\left(l_{M},i,l_{g}\right)$$

The criterion for excluding ensembles as apparent clusters for the reason of encompassing a whole dendritic segment (compare above step 3) becomes *d*_*N*_ *- d*_1_ ≥ *l*_*M*_ ≥ *d*_*N*_ *- d*_1_ *- 2 l*_*g*_ and *m* = *n*.

The overall cluster likelihood in the distance based case becomes

(21) $$\begin{array}{l} OCL_{N,n}\left(l_{m}^{*},m^{*},l_{g}\right)\\ \;=\;\sum_{l_{M}}\sum_{m=2}^{\text{min}\left(n,M\right)}SEL_{N,n}\left(l_{m},m,l_{g}\right)\cdot \delta_{N,n}\left(l_{M},m,l_{g}\right)\\ \delta_{N,n}\left(M,m,g\right)\\ \;=\{\begin{array}{cc} 1 & \;if\;SEL_{N,n}\left(l_{M},m,l_{g}\right)\leq SEL_{N,n}\left(l_{M}^{*},m^{*},l_{g}\right)\\ 0 & \begin{array}{l} \;<SEL_{N,n}\left(l_{M},m-1,l_{g}\right)\\ \text{ otherwise} \end{array} \end{array} \end{array}$$

Where the outer sum is over all possible *l*_*M*_ with the restriction that any set of spines in any interval [*d*_*i*_*, d*_*i*_ + *l*_*M*_], which satisfies more than one *l*_*M*_, is counted only once.

Equations (16) and (18) for calculating the probability of finding the number of observed dendritic segments with a synapse cluster (step 5) remain the same in the order and distance based cases. The same applies for the characterization and quantification of cluster parameters in step 6.

### Transformation to distance based treatment for branched trees

In the transformation of the analysis of branched trees (section Alternative Step 2: Calculation of the Specific Ensemble Likelihood for Branched Trees) to the distance based treatment, segments and branch points are separately dealt with again. The likelihood equation for segments can be derived by extending Equation (19) for including the *open end* condition (see example in Figure 7C):

(22) $$\begin{array}{l} p_{x}^{s}\left(l_{M},m,l_{g}\right)\\ \;=\;\left(\underset{i=1}{\sum^{N}}\left(\begin{array}{c} M_{i}^{l_{M}}\left(m\right)-2\\ m-2 \end{array}\right)\left(\begin{array}{c} N-M_{i}^{l_{M}}\left(m\right)-g_{i}^{l_{g}}-h_{i}^{l_{g}}\\ n-m \end{array}\right)\right)\frac{1}{\left(\begin{array}{c} N_{tot}\\ n_{tot} \end{array}\right)} \end{array}$$

Where the number of spines in an ensemble of length *l*_*M*_ starting with the spine at position *d*_*i*_ is as in Equation (19)

$$\begin{array}{l} M_{i}^{l_{M}}\left(m\right)\\ \;=\;\{\begin{array}{cc} \text{number of spines in} & \text{if number of spines in}\\ \left[d_{i},d_{i}+l_{M}\right] & \left[d_{i},d_{i}+l_{M}\right]\;\geq \;m\\ \; & \;\\ 0 & \text{otherwise} \end{array} \end{array}$$

and the number of spines *g* and *h* in the trailing and leading gaps of length *l*_*g*_, respectively, are as in Equation (19) for the *closed end* condition with addition of the *open end* condition:

$$\begin{array}{l} g_{i}^{l_{g}}=\{\begin{array}{cc} \text{number of spines on segment }x\text{ in }\left[0,d_{i}^{x}\right] & \text{if }d_{i}^{x}\leq l_{g}\;and\;closed\;end\\ \begin{array}{c} \;\\ \begin{array}{c} \text{sum of spines on segment }x\text{ in }\left[0,d_{i}^{x}\right],\\ \text{segment }y\text{ in }\left[d_{N}^{y}-l_{g}+d_{i}^{x},d_{N}^{y}\right]\\ \text{and segment }z\text{ in }\left[d_{N}^{z}-l_{g}+d_{i}^{x},d_{N}^{z}\right] \end{array}\\ \; \end{array} & \text{if }d_{i}^{x}\leq l_{g}\text{and }open\;end\\ \text{number of spines on segment }x\text{ in }\left[d_{i}^{x}-l_{g},d_{i}^{x}\right] & \text{if }d_{i}^{x}>l_{g} \end{array}\\ h_{i}^{l_{g}}=\{\begin{array}{cc} \begin{array}{c} \text{spines on segment }x\text{ in }\left[d_{i}^{x}+l_{M},d_{i}+l_{M}+l_{g}\right]\\ \; \end{array} & \begin{array}{c} \text{if }d_{i}^{x}+l_{M}+l_{g}<d_{N}^{x}\\ \; \end{array}\\ \text{spines on segment }x\text{ in }\left[d_{i}^{x}+l_{M},d_{N}^{x}\right] & \text{if }d_{i}^{x}+l_{M}+l_{g}\geq d_{N}^{x}\;and\;closed\\ \begin{array}{c} \;\\ \begin{array}{c} \text{sum of spines on segment }x\text{ in }\left[d_{i}^{x}+l_{M},d_{N}^{x}\right],\\ \text{segment }y\text{ in }\left[0,l_{g}-\left(d_{i}^{x}+l_{M}-d_{N}^{x}\right)\right]\\ \text{and segment }z\text{ in }\left[0,l_{g}-\left(d_{i}^{x}+l_{M}-d_{N}^{x}\right)\right] \end{array} \end{array} & \text{if }d_{i}^{x}+l_{M}+l_{g}\geq d_{N}^{x}\text{ and }open \end{array} \end{array}$$

In the *open end* condition the fraction of the length of the gap *l*_*g*_ that has to extend beyond segment *x* lies on the adjacent segments *y* and *z*. At the trailing gap this is *l*_*g*_-$d_{i}^{x}$ and at the leading gap *l*_*g*_-($d_{i}^{x}+$*l*_*M*_- $d_{N}^{x}$*)*.

The likelihood for an ensemble located at the branch point between segments *x, y*, and *z* is

(23) $$\begin{array}{l} p_{x,y,z}^{pb}\left(l_{M},m,l_{g}\right)=(\underset{i,k,j}{\sum^{N^{x},N^{y},N^{z}}}\left(\begin{array}{c} M_{i,j,k}^{l_{M}}\left(m\right)-\delta_{i,j,k}\\ m-\delta_{i,j,k} \end{array}\right)\\ \;\left(\begin{array}{c} N_{tot}-M_{i,j,k}^{l_{M}}\left(m\right)-g_{i}^{x}-g_{j}^{y}-g_{k}^{z}\\ n_{tot}-m \end{array}\right))\frac{1}{\left(\begin{array}{c} N_{tot}\\ n_{tot} \end{array}\right)} \end{array}$$

with the constraint

$$d_{i^{\prime }}^{x}+d_{j^{\prime }}^{y}+d_{k^{\prime }}^{z}\leq l_{M}$$

where the *d*′ denote spine distances from the branch point and not from the soma; with the number of spines in the ensemble

$$\begin{array}{l} M_{i,j,k}^{l_{M}}\left(m\right)=\\ \{\begin{array}{cc} \begin{array}{c} \text{sum of spines on segment}\;x\;\text{in}\left[0,d_{i^{\prime }}^{x}\right],\\ \text{segment}\;y\;\text{in}\left[0,d_{j^{\prime }}^{y}\right]\\ \text{and segment}\;z\;\text{in}\left[0,d_{k^{\prime }}^{z}\right] \end{array} & \begin{array}{c} \text{if }d_{i^{\prime }}^{x}+d_{j^{\prime }}^{y}+d_{k^{\prime }}^{z}\leq l_{M}\\ \text{and sum of spines}\geq \text{m} \end{array}\\ \; & \;\\ 0 & \text{otherwise} \end{array} \end{array}$$

with distinction of the cases where only two or all three segments at the branch point are spanned by the ensemble

$$\delta_{i,j,k}=\{\begin{array}{cc} 3 & \text{if }d_{i^{\prime }}^{x}>0\text{ and }d_{j^{\prime }}^{y}>0\text{ and }d_{k^{\prime }}^{z}>0\\ 2 & \text{otherwise} \end{array}$$

and with the spines contained in the gaps on each segment

$$\begin{array}{l} g_{i}^{x}=\text{spines on segment }x\;\text{in}\left[d_{i^{\prime }}^{x},d_{i^{\prime }}^{x}+l_{g}\right]\\ g_{j}^{y}=\text{spines on segment}\;y\;\text{in}\left[d_{j^{\prime }}^{y},d_{j^{\prime }}^{y}+l_{g}\right]\\ g_{k}^{z}=\text{spines on segment}\;z\;\text{in}\left[d_{k^{\prime }}^{z},d_{k^{\prime }}^{z}+l_{g}\right] \end{array}$$

Summation of Equations (22) and (23) over all segments and branch points, respectively, yields the likelihood to find an ensemble of a certain length with m or more inputs on the tree:

(24) $$p_{N_{tot},n_{tot}}\left(l_{M},m,l_{g}\right)=\sum_{x}p_{x}^{s}\left(l_{M},m,l_{g}\right)+\sum_{xyz}p_{x,y,z}^{pb}\left(l_{M},m,l_{g}\right)$$

Similar to Equations (11, 12) in section Alternative Step 2: Calculation of the Specific Ensemble Likelihood for Branched Trees, Equation (22) assumes that gaps do not extend beyond the directly adjacent segments in the *open end* condition and Equation (23) that the ensemble and the adjoining gaps are completely contained in the segments at the branch point and do not extend via neighboring branch points to the next segments. Algorithms for calculating the likelihoods from data should account for such cases.

In analogy to Equations (20) and (21) the *specific ensemble likelihood* within the whole tree in the *distance based case* is given by

(25) $$SEL_{N_{tot},n_{tot}}^{tree}\left(l_{M},m,l_{g}\right)=\underset{i=m}{\sum^{\min\left(n_{tot},M\right)}}p_{N_{tot},n_{tot}}\left(l_{M},i,l_{g}\right)$$

And the overall cluster likelihood becomes

(26) $$\begin{array}{l} OCL_{N_{tot},n_{tot}}^{tree}\left(l_{M}^{*},m^{*},l_{g}\right)\\ \;=\;\sum_{l_{M}}\sum_{m=2}^{\text{min}\left(n_{tot},M\right)}SEL_{N_{tot},n_{tot}}\left(l_{M},m,l_{g}\right)\cdot \delta_{N_{tot},n_{tot}}\left(l_{M},m,l_{g}\right)\\ \delta_{N_{tot},n_{tot}}\left(M,m,g\right)\\ =\;\{\begin{array}{cc} 1 & if\;SEL_{N_{tot},n_{tot}}\left(l_{M},m,l_{g}\right)\leq SEL_{N_{tot},n_{tot}}\left(l_{M}^{*},m^{*},l_{g}\right)\\ 0 & \begin{array}{l} \;<SEL_{N_{tot},n_{tot}}\left(l_{M},m-1,l_{g}\right)\\ \text{ otherwise} \end{array} \end{array} \end{array}$$

### Comparison of the combinatorial approach and the likelihood estimation by reshuffling

**Figure 8:**
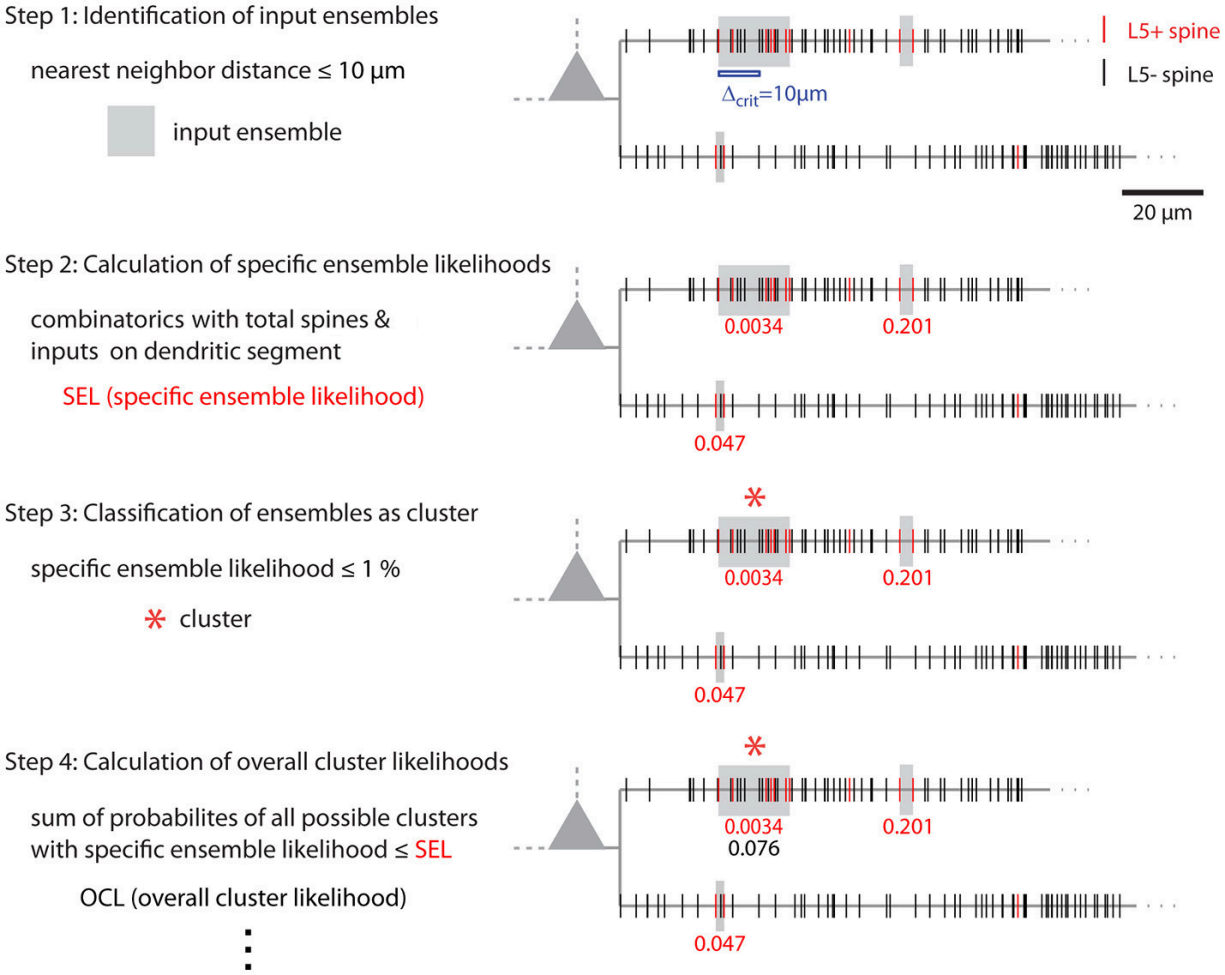
Illustration of cluster detection and analysis step 1–4. Cluster detection and analysis step 1–4 are illustrated in sequence for the dendritic segment shown on the right. Step 1 detects three ensembles (Δ_crit_ = 10 μm; gray boxes) as potential clusters. Step 2 provides the *specific ensemble likelihood* for each ensemble (red numbers). The given values were obtained with the approach for individual segments. When the whole mapped area is considered the values are SEL^tree^ = 6.8·10^−4^, 0.35, 0.26 (top semgment right, left, bottom segment). Step 3, only the ensemble nearest to the soma on the top segment has a *specific ensemble likelihood* below the criterion of 1% and is classified as cluster (red star). In step 4 the *overall cluster likelihood* for the cluster is calculated, which is used together with the overall cluster likelihoods of all clusters in the data set for statistically testing the hypothesis that overall the spatial organization of inputs is clustered (step 5, not illustrated). Finally, parameters like number of inputs, length along the dendrite etc. are determined for all clusters (step 6, not indicated). The cluster in this example contains 7 inputs and spans 17.5 μm of dendritic length.

The combinatorial approach provides an analytical solution that yields exact likelihood values. For comparison the expected error of ensemble likelihood estimates obtained by random reshuffling are provided in Figure 4B. For example, for a pattern with a likelihood of *p* = 0.01, a value that has been used to classify ensembles as clusters (see Step 3: Classification of Ensembles as Cluster According to Ensemble Likelihood Criterion), one million rounds of reshuffling are required for achieving an expected error of ±1% for the likelihood estimate. Typically only 100 to 10,000 rounds of reshuffling are performed (Takahashi et al., 2012; Yadav et al., 2012; McBride and DeBello, 2015). Such numbers of reshuffling need to be scaled up by factors of at least 100 to 10,000 to arrive at errors of ±1% or less. While increasing the number of reshuffling rounds reduces the expected error of the likelihood estimate, it increases computation time. For comparing the combinatorial approach and random reshuffling with respect to computation time, I used as example data the top right cell in Figure 1 and the single ensemble/cluster on the upper segment indicated by the gray rectangle in Figure 8. Code was programmed in MATLAB (see Supplementary Material) running on a standard desktop computer. The computation time with the presented combinatorial approach was 1.5 ± 0.2 s (*n* = 10) for determining the ensemble likelihood within the upper segment. The ensemble likelihood value is 0.0034 (Figure 8). Therefore, more than one million rounds of reshuffling are required to ensure an error of less than 1% when estimating the likelihood by reshuffling (Figure 4B, line labeled with *p* = 0.01). One million rounds of reshuffling with ensemble detection and comparison of ensemble parameters to the parameter set of interest in each round required 85 ± 1 s (~ 1.4 min; *n* = 10). The estimated likelihood value was (3.40 ± 0.07)·10^−3^ in line with the predicted error (Figure 4B). Thus in comparison, reshuffling takes a factor of about 50 longer and yields only an estimate for ensemble likelihood compared to the combinatorial approach. As another example, the analysis was performed for the same ensemble, but now for the whole mapped dendritic tree of the top right cell in Figure 1 (and see Figure 8). The combinatorial approach took 9.1 ± 0.1 s (*n* = 10) of computation time and yielded an ensemble likelihood of 6.82·10^−4^. To estimate such low likelihood with an error of less than 1% requires 10 million rounds of reshuffling. This took 1,172 ± 4 s (~20 min) of computation time and yielded a likelihood estimate of (6.62 ± 0.04)·10^−4^. In this case reshuffling took about a factor of 130 longer than the combinatorial approach. In conclusion the combinatorial approach provides a significant gain both in accuracy and computation time.

## Discussion

The spatial organization of synaptic inputs on the dendritic tree of a postsynaptic neuron is considered to play an important role in dendritic integration (Losonczy and Magee, 2006; Branco and Hausser, 2011). While the first data on the spatial arrangement of synapses on the dendrites of hippocampal or cortical pyramidal cells became available (e.g., Chen et al., 2011; da Costa and Martin, 2011; Kim et al., 2011; Kleindienst et al., 2011; Takahashi et al., 2012; Rah et al., 2013; Druckmann et al., 2014), methods for a systematic quantitative analysis of the patterns of the spatial organization of synaptic inputs on dendrites are not well developed. One type of spatial organization that is of particular interest are clusters of synaptic inputs. These might give rise to superlinear summation during synchronous activity (e.g., Mel, 1993; Larkum and Nevian, 2008; DeBello et al., 2014) and are thought to contribute to learning and memory (Govindarajan et al., 2006; DeBello, 2008; Kastellakis et al., 2015). Here I introduced an approach to determine the likelihood to observe cluster of synapses based on combinatorial analysis. The rationale is that in a random distribution of synapses, clusters of synapses occur with low probability. In contrast, if synapse cluster formation is mediated by some specific mechanism then they are expected to occur with a higher likelihood than predicted based on a random distribution. The first advantage of the combinatorial approach is that it is superior to simulations for the small likelihoods in question, because simulations require large numbers of reshuffling rounds in order to achieve sufficient accuracy and reliably for estimating small likelihoods. The second advantage is that it goes beyond current approaches for simply testing for a clustered vs. random distribution by allowing to identify and to characterize clusters. The new approach proceeds in six steps by first identifying ensembles of synapses, then calculating their likelihood predicted by a random distribution, classifying ensembles as clusters based on a likelihood threshold, calculating the likelihood to find any type of synapse cluster on the dendritic segments as predicted by a random distribution, using the latter for binomial statistics in order to test the hypotheses whether or not the inputs are indeed clustered, and finally characterizing the obtained clusters.

Published data on the spatial organization of synaptic inputs has been quantified in various ways so far. At the level of the whole dendritic tree the distribution of synapse distances from the soma has been analyzed (Rah et al., 2013; Druckmann et al., 2014) or similarly a modified Sholl analysis has been applied (Kleindienst et al., 2011). Furthermore, it was quantified whether the synapses are distributed randomly or in a structured way among dendritic segments (Druckmann et al., 2014). The approach toward the fine structure of the synapse distribution has been in general to determine the distribution of nearest neighbor synapse distances (e.g., McBride et al., 2008; Takahashi et al., 2012; Rah et al., 2013; Druckmann et al., 2014). A left shift of this distribution, i.e., toward smaller nearest neighbor distances, compared to the nearest neighbor distribution obtained by simulations with random placement, suggest clustering of synapses (Takahashi et al., 2012; Rah et al., 2013; Druckmann et al., 2014). Synapse clustering has been analyzed further by defining synapse ensembles or clusters as groups of synapses, where the nearest neighbor distances are below a certain threshold and comparing the numbers of observed clusters in the data and of expected clusters in simulated random distributions (Takahashi et al., 2012; Druckmann et al., 2014). Alternatively, a clustering index using graph theory has been calculated as ratio of the number of connected synapse pairs based on a distance criterion over all possible synapse pairs within a dendritic neighborhood (Rah et al., 2013).

However, so far no methods have been described, which allow to identify and assess individual clusters. To identify ensembles of synapses, which potentially represent clusters, I propose to use a nearest neighbor distance criterion as has been applied before (Takahashi et al., 2012; Druckmann et al., 2014). The particular value can be derived from the length scales of synaptic interactions during dendritic integration (Losonczy and Magee, 2006; Branco and Hausser, 2011) or plasticity induction (Harvey and Svoboda, 2007). Of all such ensembles, only those are classified as cluster, which would occur with a low likelihood in a random distribution. This is similar to the approach by Bendels et al. (2010) for the detection of presynaptic input sites with laser-scanning photostimulation, which assumes that one presynaptic cell leads to several neighboring “clustered” activation sites, the number of which is significantly larger than expected in the case of statistical independence. While the threshold on the ensemble likelihood for classification as cluster is in principle arbitrary, it leads to a statistically testable hypothesis by calculating the likelihood of observing any type of cluster on a specific segment. With this likelihood and binomial statistics, one can calculate the probability for finding at least the number of observed dendritic segments that carry an input cluster, among the total number of analyzed dendritic segments. This probability corresponds to the *p*-value for testing the hypotheses whether or not inputs are organized in clusters (Yadav et al., 2012). Once synapse clusters are identified, their properties such as length, number of inputs etc. can be analyzed and for example compared to the parameters reported in the context of dendritic integration (e.g., Losonczy and Magee, 2006; Branco and Hausser, 2011) or predicted by theories on synaptic plasticity during learning (Kastellakis et al., 2016).

This method can be applied to any data describing the distribution of specific anatomically or genetically defined synapses on the dendritic tree as obtained by conventional or large scale electron microscopy analysis (e.g., Bock et al., 2011; Briggman et al., 2011; da Costa and Martin, 2011; Morgan et al., 2016), GFP reconstitution across synaptic partners (GRASP, e.g., Druckmann et al., 2014), array tomography (e.g., Rah et al., 2013) or optogenetics in combination with 2-photon calcium imaging in dendritic spines (Little and Carter, 2012; Macaskill et al., 2012; Gökçe et al., 2016). Likewise, it can be applied to data on the distribution of functionally defined synapses on the postsynaptic dendrite (e.g., Chen et al., 2011; Kleindienst et al., 2011; Takahashi et al., 2012; Iacaruso et al., 2017) as well as clustered spine formation and compartmentalized synaptic plasticity during learning and experience-dependent plasticity (Makino and Malinow, 2011; Fu et al., 2012). In general, it can be applied to any other data regarding the spatial organization of structures along a one dimensional axis such as for example the distribution of presynaptic boutons along an axon (e.g., Schuemann et al., 2013).

So far simultaneous synapse mapping of two or more different types or cohorts of inputs has not been published to my knowledge. Technically this is feasible using spectral variants of Channelrhodopsin for distinguishing various inputs when combining optogenetics and calcium imaging for mapping functional synapses (Yizhar et al., 2011; Little and Carter, 2012; Macaskill et al., 2012; Klapoetke et al., 2014; Hooks et al., 2015; Gökçe et al., 2016) or spectral variants of GFP and other fluorescent markers when using GFP reconstitution across synaptic partners (GRASP, Druckmann et al., 2014; Li et al., 2016) or array tomography (Rah et al., 2013). Large-scale reconstructions by electron microscopy will also provide such data (e.g., Bock et al., 2011; Briggman et al., 2011; Morgan et al., 2016). Functional mapping the responsiveness of individual dendritic spines to specific sensory stimuli *in vivo* yields already data, where different spine cohorts are distinguished (Jia et al., 2010; Chen et al., 2011; Varga et al., 2011; Wilson et al., 2016; Iacaruso et al., 2017; Scholl et al., 2017). Thus, in the future it will be important to extend the presented approach to multiple types of synaptic inputs for quantitatively analyzing their spatial organization and mutual combination on the dendritic tree of the target neuron in order to understand the structural rules underlying the dendritic integration of multiple types of inputs.

## Author contributions

The author confirms being the sole contributor of this work and approved it for publication.

### Conflict of interest statement

The author declares that the research was conducted in the absence of any commercial or financial relationships that could be construed as a potential conflict of interest.

## References

[B1] Bassett D. S. Sporns O. (2017). Network neuroscience. Nat. Neurosci. 20, 353–364. 10.1038/nn.4502 28230844 PMC5485642

[B2] Bendels M. H. Beed P. Schmitz D. Johenning F. W. Leibold C. (2010). Detection of input sites in scanning photostimulation data based on spatial correlations. J. Neurosci. Methods 192, 286–295. 10.1016/j.jneumeth.2010.08.006 20705098

[B3] Bock D. D. Lee W. C. Kerlin A. M. Andermann M. L. Hood G. Wetzel A. W.. (2011). Network anatomy and *in vivo* physiology of visual cortical neurons. Nature 471, 177–182. 10.1038/nature09802 21390124 PMC3095821

[B4] Branco T. Hausser M. (2011). Synaptic integration gradients in single cortical pyramidal cell dendrites. Neuron 69, 885–892. 10.1016/j.neuron.2011.02.006 21382549 PMC6420135

[B5] Briggman K. L. Helmstaedter M. Denk W. (2011). Wiring specificity in the direction-selectivity circuit of the retina. Nature 471, 183–188. 10.1038/nature09818 21390125

[B6] Chen X. Leischner U. Rochefort N. L. Nelken I. Konnerth A. (2011). Functional mapping of single spines in cortical neurons *in vivo*. Nature 475, 501–505. 10.1038/nature10193 21706031

[B7] da Costa N. M. Martin K. A. (2011). How thalamus connects to spiny stellate cells in the cat's visual cortex. J. Neurosci. 31, 2925–2937. 10.1523/JNEUROSCI.5961-10.2011 21414914 PMC6623786

[B8] DeBello W. M. (2008). Micro-rewiring as a substrate for learning. Trends Neurosci. 31, 577–584. 10.1016/j.tins.2008.08.006 18817991 PMC2581897

[B9] DeBello W. M. Mcbride T. J. Nichols G. S. Pannoni K. E. Sanculi D. Totten D. J. (2014). Input clustering and the microscale structure of local circuits. Front. Neural Circuits 8:112. 10.3389/fncir.2014.00112 25309336 PMC4162353

[B10] Druckmann S. Feng L. Lee B. Yook C. Zhao T. Magee J. C.. (2014). Structured synaptic connectivity between hippocampal regions. Neuron 81, 629–640. 10.1016/j.neuron.2013.11.026 24412418

[B11] Fu M. Yu X. Lu J. Zuo Y. (2012). Repetitive motor learning induces coordinated formation of clustered dendritic spines *in vivo*. Nature 483, 92–95. 10.1038/nature10844 22343892 PMC3292711

[B12] Gökçe O. Bonhoeffer T. Scheuss V. (2016). Clusters of synaptic inputs on dendrites of layer 5 pyramidal cells in mouse visual cortex. Elife 5:e09222. 10.7554/eLife.09222 27431612 PMC4951190

[B13] Govindarajan A. Kelleher R. J. Tonegawa S. (2006). A clustered plasticity model of long-term memory engrams. Nat. Rev. Neurosci. 7, 575–583. 10.1038/nrn1937 16791146

[B14] Harvey C. D. Svoboda K. (2007). Locally dynamic synaptic learning rules in pyramidal neuron dendrites. Nature 450, 1195–1200. 10.1038/nature06416 18097401 PMC3425382

[B15] Hooks B. M. Lin J. Y. Guo C. Svoboda K. (2015). Dual-channel circuit mapping reveals sensorimotor convergence in the primary motor cortex. J. Neurosci. 35, 4418–4426. 10.1523/JNEUROSCI.3741-14.2015 25762684 PMC4355205

[B16] Iacaruso M. F. Gasler I. T. Hofer S. B. (2017). Synaptic organization of visual space in primary visual cortex. Nature 547, 449–452. 10.1038/nature23019 28700575 PMC5533220

[B17] Jia H. Rochefort N. L. Chen X. Konnerth A. (2010). Dendritic organization of sensory input to cortical neurons *in vivo*. Nature 464, 1307–1312. 10.1038/nature08947 20428163

[B18] Kastellakis G. Cai D. J. Mednick S. C. Silva A. J. Poirazi P. (2015). Synaptic clustering within dendrites: an emerging theory of memory formation. Progr. Neurobiol. 126, 19–35. 10.1016/j.pneurobio.2014.12.002 25576663 PMC4361279

[B19] Kastellakis G. Silva A. J. Poirazi P. (2016). Linking memories across time via neuronal and dendritic overlaps in model neurons with active dendrites. Cell Rep. 17, 1491–1504. 10.1016/j.celrep.2016.10.015 27806290 PMC5149530

[B20] Kim J. Zhao T. Petralia R. S. Yu Y. Peng H. Myers E.. (2011). mGRASP enables mapping mammalian synaptic connectivity with light microscopy. Nat. Methods 9, 96–102. 10.1038/nmeth.1784 22138823 PMC3424517

[B21] Klapoetke N. C. Murata Y. Kim S. S. Pulver S. R. Birdsey-Benson A. Cho Y. K. (2014). Independent optical excitation of distinct neural populations. Nat. Meth. 11, 338–346. 10.1038/nmeth.2836 PMC3943671 24509633

[B22] Kleindienst T. Winnubst J. Roth-Alpermann C. Bonhoeffer T. Lohmann C. (2011). Activity-dependent clustering of functional synaptic inputs on developing hippocampal dendrites. Neuron 72, 1012–1024. 10.1016/j.neuron.2011.10.015 22196336

[B23] Larkum M. E. Nevian T. (2008). Synaptic clustering by dendritic signalling mechanisms. Curr. Opin. Neurobiol. 18, 321–331. 10.1016/j.conb.2008.08.013 18804167

[B24] Lavzin M. Rapoport S. Polsky A. Garion L. Schiller J. (2012). Nonlinear dendritic processing determines angular tuning of barrel cortex neurons *in vivo*. Nature 490, 397–401. 10.1038/nature11451 22940864

[B25] Li Y. Guo A. Li H. (2016). CRASP: CFP reconstitution across synaptic partners. Biochem. Biophys. Res. Commun. 469, 352–356. 10.1016/j.bbrc.2015.12.011 26682922

[B26] Little J. P. Carter A. G. (2012). Subcellular synaptic connectivity of layer 2 pyramidal neurons in the medial prefrontal cortex. J. Neurosci. 32, 12808–12819. 10.1523/JNEUROSCI.1616-12.2012 22973004 PMC3490687

[B27] Losonczy A. Magee J. C. (2006). Integrative properties of radial oblique dendrites in hippocampal CA1 pyramidal neurons. Neuron 50, 291–307. 10.1016/j.neuron.2006.03.016 16630839

[B28] Macaskill A. F. Little J. P. Cassel J. M. Carter A. G. (2012). Subcellular connectivity underlies pathway-specific signaling in the nucleus accumbens. Nat. Neurosci. 15, 1624–1626. 10.1038/nn.3254 23143514 PMC3986679

[B29] Makino H. Malinow R. (2011). Compartmentalized versus global synaptic plasticity on dendrites controlled by experience. Neuron 72, 1001–1011. 10.1016/j.neuron.2011.09.036 22196335 PMC3310180

[B30] McBride T. J. DeBello W. M. (2015). Input clustering in the normal and learned circuits of adult barn owls. Neurobiol. Learn. Mem. 121, 39–51. 10.1016/j.nlm.2015.01.011 25701706 PMC4465939

[B31] McBride T. J. Rodriguez-Contreras A. Trinh A. Bailey R. Debello W. M. (2008). Learning drives differential clustering of axodendritic contacts in the barn owl auditory system. J. Neurosci. 28, 6960–6973. 10.1523/JNEUROSCI.1352-08.2008 18596170 PMC2581896

[B32] Mel B. W. (1993). Synaptic integration in an excitable dendritic tree. J. Neurophysiol. 70, 1086–1101. 10.1152/jn.1993.70.3.1086 8229160

[B33] Morgan J. L. Berger D. R. Wetzel A. W. Lichtman J. W. (2016). The fuzzy logic of network connectivity in mouse visual thalamus. Cell 165, 192–206. 10.1016/j.cell.2016.02.033 27015312 PMC4808248

[B34] Poirazi P. Mel B. W. (2001). Impact of active dendrites and structural plasticity on the memory capacity of neural tissue. Neuron 29, 779–796. 10.1016/S0896-6273(01)00252-5 11301036

[B35] Rah J.-C. Bas E. Colonell J. Mishchenko Y. Karsh B. Fetter R. D.. (2013). Thalamocortical input onto layer 5 pyramidal neurons measured using quantitative large-scale array tomography. Front. Neural Circuits 7:177. 10.3389/fncir.2013.00177 24273494 PMC3824245

[B36] Scholl B. Wilson D. E. Fitzpatrick D. (2017). Local order within global disorder: synaptic architecture of visual space. Neuron 96, 1127.e1124–1138.e1124. 10.1016/j.neuron.2017.10.017 29103806 PMC5868972

[B37] Schroter M. Paulsen O. Bullmore E. T. (2017). Micro-connectomics: probing the organization of neuronal networks at the cellular scale. Nat. Rev. Neurosci. 18, 131–146. 10.1038/nrn.2016.182 28148956

[B38] Schuemann A. Klawiter A. Bonhoeffer T. Wierenga C. J. (2013). Structural plasticity of GABAergic axons is regulated by network activity and GABAA receptor activation. Front. Neural Circuits 7:113. 10.3389/fncir.2013.00113 23805077 PMC3693093

[B39] Smith S. L. Smith I. T. Branco T. Hausser M. (2013). Dendritic spikes enhance stimulus selectivity in cortical neurons *in vivo*. Nature 503, 115–120. 10.1038/nature12600 24162850 PMC6319606

[B40] Takahashi N. Kitamura K. Matsuo N. Mayford M. Kano M. Matsuki N.. (2012). Locally synchronized synaptic inputs. Science 335, 353–356. 10.1126/science.1210362 22267814

[B41] Varga Z. Jia H. Sakmann B. Konnerth A. (2011). Dendritic coding of multiple sensory inputs in single cortical neurons *in vivo*. Proc. Natl. Acad. Sci. U.S.A. 108, 15420–15425. 10.1073/pnas.1112355108 21876170 PMC3174623

[B42] Wilson D. E. Whitney D. E. Scholl B. Fitzpatrick D. (2016). Orientation selectivity and the functional clustering of synaptic inputs in primary visual cortex. Nat. Neurosci. 19, 1003–1009. 10.1038/nn.4323 27294510 PMC5240628

[B43] Yadav A. Gao Y. Z. Rodriguez A. Dickstein D. L. Wearne S. L. Luebke J. I.. (2012). Morphologic evidence for spatially clustered spines in apical dendrites of monkey neocortical pyramidal cells. J. Comp. Neurol. 520, 2888–2902. 10.1002/cne.23070 22315181 PMC3573331

[B44] Yizhar O. Fenno L. E. Prigge M. Schneider F. Davidson T. J. O'shea D. J.. (2011). Neocortical excitation/inhibition balance in information processing and social dysfunction. Nature 477, 171–178. 10.1038/nature10360 21796121 PMC4155501

