# Supplementary material for: Quantitative Analysis of the Spatial Organization of Synaptic Inputs on the Postsynaptic Dendrite
Source: Front Neural Circuits. 2018 May 23;12:39. doi: 10.3389/fncir.2018.00039 (PMC5974225; doi:10.3389/fncir.2018.00039)
Supplement: Supplementary file 1 [file Data_Sheet_1.PDF]

frontiers

Supplementary Material

Quantitative Analysis of the Spatial Organization of Synaptic Inputs on the Postsynaptic Dendrite

Volker Scheuss*

*** Correspondence:** scheuss@neuro.mpg.de

1. Supplementary Code

Code in Matlab (MathWorks) for analyzing the spatial organization of synapses on the dendrite as described. In addition, code for generating synapse patterns for exploring the approach with simulated data and for estimating ensemble likelihoods by random reshuffling.

1.1. Code for generating a vector of synapse positions with uniform nearest neighbor distance

```
function distanceData=vs_uniform_distances_segment_generator(N_total)
%12/10/17 - Volker Scheuss
%INPUT
%N_total - total number of synapses (= synapse positions)
%OUTPUT
%distanceData - vector of synapse positions

%distance increasing in steps of '1'
distanceData=[0:1:N_total];
```

1.2. Code for generating a vector of synapse positions with variable nearest neighbor distance

```
function
distanceData=vs_nonuniform_distances_segment_generator(N_total,distanceMean,distanceS
td)
%12/10/17 - Volker Scheuss
%INPUT
%N_total - total number of synapses (= synapse positions)
%distanceMean - mean of distributon of nearest neighbor synapse distances
%distanceStd - standard deviation of distributon of nearest neighbor synapse
distances
%OUTPUT
%distanceData - vector of synapse positions

%random symapling of distance distribution
distanceDistribution=distanceMean+distanceStd.*randn(N_total,1);
tempIndex=find(distanceDistribution<0);
distanceDistribution(tempIndex)=0;
```

```
distanceData(1)=0;
for i=2:N_total
    distanceData(i)=distanceData(i-1)+distanceDistribution(i);
end
```

1.3. Code for randomly assigning inputs to synapses

```
function patternData=vs_random_pattern_generator(N_total,n_inputs)
%12/10/17 - Volker Scheuss
%INPUT
%N_total - total number of synapses (= synapse positions)
%n_inputs - total number of specific inputs
%OUTPUT
%patternData - vector of zeros and ones corresponding to absence or
%presence of the specific input at the particular synapse position,
%respectively

%generation of vector of random positions contained in IX
[val,IX]=sort(rand(N_total,1));
%generate vector of all N_total synapse positions set to '0' (= no specific input)
temp=zeros(1,N_total);
%set positions indexed by the first the first n_input entries of IX to '1'
%(=location with specific input)
temp(IX(1:n_inputs))=1;
%assign output
patternData=temp;
```

1.4. Code for displaying spatial organization of synapses and inputs

```
function vs_plot_segmentData(patternData,distanceData)
%13/10/17 - Volker Scheuss
%INPUT
%patternData - vector of zeros and ones corresponding to absence or
%presence of the specific input at the particular synapse position,
%respectively
%distanceData - vector of synapse positions

temp=find(patternData==1)

figure;hold;
h=plot(distanceData,ones(size(distanceData,2),1))
set(h,'Marker','+')
h=plot(distanceData(temp),ones(size(temp,2),1),'red')
set(h,'Marker','+','Linestyle','none')
```

1.5. Code for pattern analysis

```
%12/4/18 - Volker Scheuss (previous version 12/10/17)
%INPUT
%patternData - vector of zeros and ones (ones for spines with input)
%distanceData - vector of distance values describing spine positions along the
dendrite (same size as vector 'patternData')
%distanceCriterion - single value describing largest distance between nearest neigbor
spines with input as criterion to participate in a cluster
%OUTPUT
%output - structure containing values of ensemble likelihood, specific
%cluster likelihood, etc.

% identify clusters and generate table
result=analysisDistanceCriterion2(patternData,distanceData,distanceCriterion);
output.clusterData=result;

% determine cluster likelihoods
if output.clusterData.clusterExistence==1

tempLikelihoodTable=likelyhoodDistanceCombinatoricsTotal3(distanceData,output.cluster
Data.totalRespSpines,distanceCriterion);
    result=[];
    result2=[];
    for  i=1:size(output.clusterData.clusterLength,2)

result(i)=likelyhoodDistanceCombinatorics(distanceData,output.clusterData.clusterLeng
th(i),distanceCriterion,...

output.clusterData.clusterRespSpines(i),output.clusterData.totalRespSpines);

result2(i)=likelihoodTableAnalysis_vs2(tempLikelihoodTable,result(i),distanceCriterio
n,output.clusterData.totalRespSpines);
    end
    output.clusterLikelihood=result;
    output.clusterLikelihoodAny=result2;
else
    output.clusterLikelihood=[];
    output.clusterLikelihoodAny=[];
end
end

%-----------------------------------------------------------
%local functions
%-----------------------------------------------------------
function result=analysisDistanceCriterion2(patternData,distanceData,criterion)
%basic pattern parameters, identification of clusters
%created vs 01/11/12
%mod vs 29/04/13

indicesPositive=find(patternData);
result.totalRespSpines=size(indicesPositive,2);
result.segmSpines=size(patternData,2);
result.segmLength=distanceData(end)-distanceData(1);
if ~isempty(indicesPositive)==1 %if there are spines with input
    dist=distanceData(indicesPositive);
```

```
    diffDist=diff(dist); %distances between consecutive spines with input
    indDiffDist=find(diffDist<criterion); %indices of those distances, which are
below distance criterion
    if isempty(indDiffDist)==1
        result.clusterExistence=0;
    else
        result.clusterExistence=1;
        posPattern=zeros(size(diffDist,2),1);
        posPattern(indDiffDist)=1; %vector zeros and ones, where distance is below
distance criterion
        diffPosPattern=diff(posPattern); %difference vector, where '1' marks start
and '-1' end of clusters, shifted by 1
        indexStart=find(diffPosPattern==1)+1; %start indices of all clusters
        if posPattern(1)==1 %if 1st cluster starts at 1st spine, this need to be
appended
            indexStart=[1; indexStart];
        end
        indexEnd=find(diffPosPattern==-1)+1; %end indices of all clusters
        if posPattern(end)==1 %if last cluster ends at last spine, this need to be
appended
            indexEnd=[indexEnd; size(posPattern,1)+1];
        end
        result.cluster1stSpine=indicesPositive(indexStart); %indices of 1st spine in
all cluster (i.e. left hand boundary)
        result.clusterLastSpine=indicesPositive(indexEnd); %indices of last spine in
all cluster (i.e. right hand boundary)
        result.posPattern=posPattern;
        %cluster parameters
        for i=1:size(indexStart,1)
            result.clusterLength(i)=distanceData(result.clusterLastSpine(i))-
distanceData(result.cluster1stSpine(i));

tempPatternData=patternData(result.cluster1stSpine(i):result.clusterLastSpine(i));
            result.clusterNegSpines(i)=-sum(tempPatternData-1);
            result.clusterRespSpines(i)=sum(tempPatternData);

result.clusterTotSpines(i)=result.clusterNegSpines(i)+result.clusterRespSpines(i);

result.clusterPackRatio(i)=result.clusterRespSpines(i)/result.clusterTotSpines(i);
        end
        %gap parameters
        if size(indexStart,1)==1 %if only one cluster, gap parameters are set 'empty'
- BETTER put data for leading/trailing 'gap'?
            result.gapLength=[];
            tempPatternData=[];
            result.gapNegSpines=[];
            result.gapRespSpines=[];
            result.gapTotSpines=[];
            result.gapPackRatio=[];
        else
            if ~(result.cluster1stSpine(1)==1) %if 1st cluster does not start with
1st spine in segment
                result.gapLength(1)=distanceData(result.cluster1stSpine(1))-
distanceData(1);
                tempPatternData=patternData(1:result.cluster1stSpine(1));
                result.gapNegSpines(1)=-sum(tempPatternData-1);
                result.gapRespSpines(1)=sum(tempPatternData)-1;
```

```
result.gapTotSpines(1)=result.gapNegSpines(1)+result.gapRespSpines(1);

result.gapPackRatio(1)=result.gapRespSpines(1)/result.gapTotSpines(1);
                a=1;
            else
                a=0;
            end
            %all gaps between 1st and last cluster
            for i=1:size(indexStart,1)-1
                result.gapLength(i+a)=distanceData(result.cluster1stSpine(i+1))-
distanceData(result.clusterLastSpine(i));

tempPatternData=patternData(result.clusterLastSpine(i):result.cluster1stSpine(i+1));
                result.gapNegSpines(i+a)=-sum(tempPatternData-1);
                result.gapRespSpines(i+a)=sum(tempPatternData)-2;

result.gapTotSpines(i+a)=result.gapNegSpines(i+a)+result.gapRespSpines(i+a);

result.gapPackRatio(i+a)=result.gapRespSpines(i+a)/result.gapTotSpines(i+a);
            end
            if ~(result.clusterLastSpine(end)==size(patternData,2)) %if last cluster
does not end with last spine in segment
                i=i+a+1;
                result.gapLength(i)=distanceData(end)-
distanceData(result.clusterLastSpine(end));
                tempPatternData=patternData(result.clusterLastSpine(end):end);
                result.gapNegSpines(i)=-sum(tempPatternData-1);
                result.gapRespSpines(i)=sum(tempPatternData)-1;

result.gapTotSpines(i)=result.gapNegSpines(i)+result.gapRespSpines(i);

result.gapPackRatio(i)=result.gapRespSpines(i)/result.gapTotSpines(i);
            end
        end
    end
else
    result.clusterExistence=0;
end
end

%---------------------------------------------------------------------------
function
result=likelyhoodDistanceCombinatoricsTotal3(distances,totalRespSpines,gapLength)
%creates likelihood table used as input to function 'likelihoodTableAnalysis_vs2'

totalSpines=size(distances,2);
distances=distances-distances(1);

counter=0;

total_combinations=prod([totalSpines-totalRespSpines+1:1:totalSpines])/...
    prod([1:1:totalRespSpines]);
for i=1:size(distances,2)
    for j=1:size(distances,2)-i
        counter=counter+1;
        patchTable(i).patchLength(j)=distances(i+j)-distances(i);
        patchTable(i).patchSpines(j)=j+1;
```

```
        %leading spines outside gap
        if (distances(i)-gapLength)>0
            tempIndex=find(distances<(distances(i)-gapLength));
            patchTable(i).leadingSpines=size(tempIndex,2);
        else
            patchTable(i).leadingSpines=0;
        end
        %trailing spines outside gap
        if (distances(i)+gapLength)<distances(end)
            tempIndex=find(distances>(distances(i+j)+gapLength));
            patchTable(i).trailingSpines(j)=size(tempIndex,2);
        else
            patchTable(i).trailingSpines(j)=0;
        end
        %likelihoods
        if patchTable(i).patchSpines(j)>totalRespSpines
            loopEnd=totalRespSpines;
        else
            loopEnd=patchTable(i).patchSpines(j);
        end
        for k=2:loopEnd
            if patchTable(i).leadingSpines+patchTable(i).trailingSpines(j) >
totalRespSpines-k
                patchTable(i).likelihood(j,k-1)=...

nchoosek(patchTable(i).leadingSpines+patchTable(i).trailingSpines(j),totalRespSpines-
k)*...
                    nchoosek(patchTable(i).patchSpines(j)-2,k-2)/...
                    total_combinations;
            else
                patchTable(i).likelihood(j,k-1)=0;
            end
        end
    end
end
result=patchTable;
end
%---------------------------------------------------------------------------
function
result=likelyhoodDistanceCombinatorics(distances,clusterLength,gapLength,clusterRespS
pines,totalRespSpines)
%likelihood for cluster type (cluster length, minimum of responsive spines)

totalSpines=size(distances,2);

distances=distances-distances(1); %distances(1,2)? -changed to distances(1) VS31/1/13
occurences=0;
for i=1:size(distances,2)

clusterIndex=find((distances>=distances(i))==(distances<=(distances(i)+clusterLength)
)); %finds for spine at index(i) all spines within patch of size clusterlength
    clusterNum=size(clusterIndex,2); %number of spines in patch with clusterlength at
position i
    if clusterNum>=clusterRespSpines
        leadingGapIndex=find((distances>=(distances(i)-
gapLength))==(distances<distances(i)));
        leadingGapNum=size(leadingGapIndex,2); %number of spines in leading gap
```

```
trailingGapIndex=find((distances>(distances(i)+clusterLength))==(distances<(distances
(i)+clusterLength+gapLength)));
        trailingGapNum=size(trailingGapIndex,2); %number of spines in trailing gap
        if clusterNum>totalRespSpines %check if loop has to run over all responsive
spines or maximal spines fitting into patch
            loopEnd=totalRespSpines;
        elseif clusterNum<=totalRespSpines
            loopEnd=clusterNum;
        end
        for j=clusterRespSpines:loopEnd
            if totalSpines-clusterNum-leadingGapNum-trailingGapNum >=totalRespSpines-
j
                occurences=occurences+nchoosek(clusterNum-2,j-2)*...
                    nchoosek(totalSpines-clusterNum-leadingGapNum-
trailingGapNum,totalRespSpines-j);
            end
        end
    end

end
total_combinations=prod([totalSpines-totalRespSpines+1:1:totalSpines])/...
    prod([1:1:totalRespSpines]);
result=occurences/total_combinations;

end

%---------------------------------------------------------------------------
function
result=likelihoodTableAnalysis_vs2(patchTable,criterion,gapLength,totalRespSpines)
%likelihood to finde any cluster in segment with cluster likelyhodd <= criterion

counter=1;
%initialization of "participation" field
for i=1:size(patchTable,2)
    if patchTable(i).patchSpines(end)<totalRespSpines

patchTable(i).participation=zeros(size(patchTable(i).patchLength,2),patchTable(i).pat
chSpines(end)-1);
    else

patchTable(i).participation=zeros(size(patchTable(i).patchLength,2),totalRespSpines-
1);
    end
end

for i=1:size(patchTable,2)
    for j=1:size(patchTable(i).patchLength,2)
        tempPatchLength=patchTable(i).patchLength(j);
        %minimal filling
        minSpines=ceil(tempPatchLength/gapLength); %table starts with 1+1 spine
        if minSpines==0 %neighbors with 0 distance
            minSpines=1;
        end
        %maximal filling
        if patchTable(i).patchSpines(j)<=totalRespSpines
            maxSpines=patchTable(i).patchSpines(j)-1; %table starts with 1+1 spine
        else
```

```
            maxSpines=totalRespSpines-1; %table starts with 1+1 spine
        end
        %length index
                for k=1:size(patchTable,2)
            lengthIndexTemp=find(patchTable(k).patchLength<=tempPatchLength);
            if isempty(lengthIndexTemp)
                lengthIndex(k)=0;
            else
                lengthIndex(k)=lengthIndexTemp(end);
            end
        end
        %likelihood determination
        tempLikelihood=0;
        tempNumSpines=maxSpines;
        kIncluded=[];
        flag=1;
        while flag==1
            tempLikelihood2=0;
            tempKincluded=[];
            for k=1:size(patchTable,2)
                if lengthIndex(k)>0
                    if size(patchTable(k).likelihood,2)>=tempNumSpines

tempLikelihood2=tempLikelihood2+patchTable(k).likelihood(lengthIndex(k),tempNumSpines
);
                        tempKincluded=[tempKincluded; k lengthIndex(k)
tempNumSpines];
                    end
                end
            end
            tempNumSpines=tempNumSpines-1;
            if tempLikelihood+tempLikelihood2>criterion
                flag=0;
            else
                tempLikelihood=tempLikelihood+tempLikelihood2;
                kIncluded=[kIncluded; tempKincluded];
            end
            if tempNumSpines<minSpines
                flag=0;
            end
        end
        if tempLikelihood>0
            likelihood.value(counter)=tempLikelihood;
            for k=1:size(kIncluded,1)

patchTable(kIncluded(k,1)).participation(kIncluded(k,2),kIncluded(k,3))=1;
            end
            counter=counter+1;
        end
    end
end

for i=1:size(patchTable,2)
    tempLikelihood=patchTable(i).likelihood.*patchTable(i).participation;
    overAlllikelihood(i)=sum(tempLikelihood(:));
end
result=sum(overAlllikelihood(:));
```

```
if result>1
    result=1;
end

end
%------------------------------------------------------------------------------
```

1.6.Code for detecting ensembles with specified ensemble parameters (called by function in point 1.7.)

```
function detected=vs_ensemble_detection(patternData,distanceData,...
    ensembleCriterion,ensembleLength,ensembleSynapses,ensembleInputs)
%29/03/17 - Volker Scheuss
%INPUT
%patternData - vector of zeros and ones corresponding to absence or
%presence of the specific input at the particular synapse position,
%respectively
%distanceData - vector of synapse positions
%ensembleLength - length of ensemble
%ensembleSynapses - minimum number of synapses in the ensemble
%ensembleInputs - minimum number of inputs in ensemble

temp=find(patternData==1);
distanceDataInputs=distanceData(temp);
distanceDataInputsDiff=diff(distanceDataInputs);

ensembleFlag=temp*0;
ensembleFlag(1)=1;
for i=1:size(ensembleFlag,2)-1
    if distanceDataInputsDiff(i) <= ensembleCriterion
        ensembleFlag(i+1)=ensembleFlag(i);
    else
        ensembleFlag(i+1)=ensembleFlag(i)+1;
    end
end

ensemblesInputs=[];
ensemblesM=[];
ensemblesLength=[];
for i=1:ensembleFlag(end)
    ensembleIndices=find(ensembleFlag==i);
    ensemblesInputs(i)=size(ensembleIndices,2);
    ensemblesM(i)=temp(ensembleIndices(end))-temp(ensembleIndices(1))+1;
    ensemblesLength(i)=distanceData(temp(ensembleIndices(end)))-...
        distanceData(temp(ensembleIndices(1)));
end

detected=0;
for i=1:ensembleFlag(end)
    if ensemblesLength(i) <= ensembleLength
        if ensemblesInputs(i) >= ensembleInputs
            if ensemblesM(i) >= ensembleSynapses
                detected=1;
            end
        end
    end
```

```
end
```

1.7.Code for estimating ensemble likelihood by random reshuffling

```
function [likelihood timeStop]=vs_ensemble_detection_shuffling(numInputs,...
    distanceData,...
    ensembleCriterion,ensembleLength,ensembleSynapses,ensembleInputs,runs)
%29/03/17 - Volker Scheuss
%INPUT
%numInputs - number of inputs on segment
%distanceData - vector of synapse positions
%ensembleLength - length of ensemble
%ensembleSynapses - minimum number of synapses in the ensemble
%ensembleInputs - minimum number of inputs in ensembl
%runs - number of reshuffling trials

numSynapses=size(distanceData,2);
tic;
detected=[];
for i=1:runs
    patternData=vs_random_pattern_generator(numSynapses,numInputs);
    detected(i)=vs_ensemble_detection(patternData,distanceData,...
    ensembleCriterion,ensembleLength,ensembleSynapses,ensembleInputs);
end

timeStop=toc;
likelihood=sum(detected)/runs;
```
